# Modeling of the Human Alveolar Rhabdomyosarcoma *Pax3-Foxo1* Chromosome Translocation in Mouse Myoblasts Using CRISPR-Cas9 Nuclease

**DOI:** 10.1371/journal.pgen.1004951

**Published:** 2015-02-06

**Authors:** Irina V. Lagutina, Virginia Valentine, Fabrizio Picchione, Frank Harwood, Marcus B. Valentine, Barbara Villarejo-Balcells, Jaime J. Carvajal, Gerard C. Grosveld

**Affiliations:** 1 Departments of Genetics, St. Jude Children’s Research Hospital, Memphis, Tennessee, United States of America; 2 Tumor Cell Biology, St. Jude Children’s Research Hospital, Memphis, Tennessee, United States of America; 3 Division of Cancer Biology, The Institute of Cancer Research, London, United Kingdom; 4 Centro Andaluz de Biología del Desarrollo (CSIC/UPO/JA), Sevilla, Spain; University of Washington, UNITED STATES

## Abstract

Many recurrent chromosome translocations in cancer result in the generation of fusion genes that are directly implicated in the tumorigenic process. Precise modeling of the effects of cancer fusion genes in mice has been inaccurate, as constructs of fusion genes often completely or partially lack the correct regulatory sequences. The reciprocal t(2;13)(q36.1;q14.1) in human alveolar rhabdomyosarcoma (A-RMS) creates a pathognomonic *PAX3-FOXO1* fusion gene. *In vivo* mimicking of this translocation in mice is complicated by the fact that *Pax3* and *Foxo1* are in opposite orientation on their respective chromosomes, precluding formation of a functional *Pax3-Foxo1* fusion via a simple translocation. To circumvent this problem, we irreversibly inverted the orientation of a 4.9 Mb syntenic fragment on chromosome 3, encompassing *Foxo1*, by using *Cre*-mediated recombination of two pairs of unrelated oppositely oriented LoxP sites situated at the borders of the syntenic region. We tested if spatial proximity of the *Pax3* and *Foxo1* loci in myoblasts of mice homozygous for the inversion facilitated *Pax3-Foxo1* fusion gene formation upon induction of targeted CRISPR-Cas9 nuclease-induced DNA double strand breaks in *Pax3* and *Foxo1*. Fluorescent *in situ* hybridization indicated that fore limb myoblasts show a higher frequency of *Pax3/Foxo1* co-localization than hind limb myoblasts. Indeed, more fusion genes were generated in fore limb myoblasts via a reciprocal t(1;3), which expressed correctly spliced *Pax3-Foxo1* mRNA encoding Pax3-Foxo1 fusion protein. We conclude that locus proximity facilitates chromosome translocation upon induction of DNA double strand breaks. Given that the *Pax3-Foxo1* fusion gene will contain all the regulatory sequences necessary for precise regulation of its expression, we propose that CRISPR-Cas9 provides a novel means to faithfully model human diseases caused by chromosome translocation in mice.

## Author Summary

Many cancers carry recurrent chromosome translocations, which often result in the formation of fusion genes that are directly involved in the tumorigenic process. Alveolar rhabdomyosarcoma, a muscle tumor in children, is typified by a translocation that fuses the *PAX3* gene on chromosome 2 to the *FOXO1* gene on chromosome 13. For translocation to occur both genes need to break and the disparate ends need to fuse via a process called non-homologous end joining. We determined that physical proximity of *Pax3* and *Foxo1* in mouse muscle progenitor cells (myoblasts) facilitates fusion gene formation. Because *Pax3* and *Foxo1* in the mouse are in an opposite orientation, we used a chromosome engineering strategy to invert the orientation of *Foxo1* so that upon translocation a productive *Pax3-Foxo1* fusion gene is created. Co-localization of the *Pax3* and *Foxo1* loci is higher in fore limb than in hind limb myoblasts. Simultaneous induction of a targeted double strand DNA break in each gene by CRISPR-Cas9 nuclease generated more fusion genes in fore limb than in hind limb myoblasts. Thus, gene proximity facilitates fusion gene formation. We propose that CRISPR-Cas9 nuclease can be used for the precise modeling of chromosome translocations of human cancer in mice.

## Introduction

Rhabdomyosarcoma (RMS) is the third most common soft-tissue sarcoma in children with an annual incidence of five new cases per million. It accounts for 5–8% of all pediatric cancer. RMS belongs to the family of small round blue cell tumors of childhood and exhibits histological features of skeletal muscle. Two major histological subtypes of RMS can be distinguished, embryonal (E-RMS) and alveolar (A-RMS). E-RMS has its highest incidence in infants and young children whereas A-RMS is more frequent in older children and adolescents. A-RMS has a more aggressive clinical behavior with early dissemination, a poor response to chemotherapy, frequent relapses, and a 5-year failure-free survival of 65% after treatment [1]. A-RMS is found predominantly in the extremities (42%), parameningeal (17%), head and neck (11%) and other locations (21%) [1] including the trunk, perirectal and perianal areas [2, 3]. Cytogenetically A-RMS is distinguished from E-RMS by one of two recurrent chromosome translocations: t(2;13) or t(1;13), which result in fusion of *PAX3* or *PAX7* to *FOXO1*, respectively [4].

In spite of multiple attempts to identify the cell of origin in which the t(2;13) occurs the question remains unanswered. It was shown previously that transcription occurs at a few hundred discrete nuclear sites called transcription factories [5]. Some genes frequently involved in a recurrent chromosome translocation (*MYC* and *IGH* in B lymphoid progenitors, *TMPRSS2* and *ERG* or *ETV1* in prostate cancer, *RET* and *H4* in in radiation-associated papillary thyroid cancer) co-localize to the same transcription factory [6–9]. Initial chromosome conformation capture experiments in activated mouse B cells suggested that physical proximity of the *IGH* and *MYC* loci is a minor contributor to the frequency of chromosome translocation [10]. However, combined high resolution Hi-C mapping and genome-wide translocation sequencing in transformed mouse pre-B cells found good coincidence between chromosomal translocation and spatial proximity [11]. A possible driver of double strand DNA breaks might be the co-localization of replication stress-induced early replication fragile sites (ERFSs) with highly expressed gene clusters [12]. Though it was demonstrated that ectopic expression of PAX3-FOXO1/Pax3-Foxo1 can transform mouse mesenchymal stem cells *in vitro* [13] as well as Myf6^+^ myofibers *in vivo* [14] in view of the above these cell types seem unlikely hosts for the chromosome translocation given that they do not express Pax3. In fact, the suggestion that Myf6^+^ myofibers might be the host of the PAX3-FOXO translocation was recently rectified [15].

In contrast, Pax3 is expressed in activated myoblasts upon muscle injury or in growing muscles during normal development [16]. Moreover, PAX3-FKHR, in cooperation with loss of p16^INK4A^ expression, transforms both fetal and postnatal primary human skeletal muscle cell precursors [17]. Together these observations suggest that translocation might occur in a population of activated myoblasts that express PAX3 (PAX3^+^). It has been shown that Pax3 expression differs among different muscles in the mouse [18, 19]. There are many more Pax3^+^ cells in fore limb than in hind limb muscles [19]. Muscle satellite cells from the masseter and soleus did not express Pax3 while only 7% of those from the *extensor digitorum longus* (EDL) did. In contrast 49% of satellite cells from the biceps were Pax3^+^. In addition, most ventral trunk muscles were Pax3-positive and 64% of satellite cells from the diaphragm expressed Pax3. Importantly, primary myoblast cultures of Pax3^+^ satellite cells remain Pax3^+^, while Pax3^-^ satellite cells from hind limb remain negative [19].

Studies addressing the relation between spatial chromosome proximity and translocation have been performed in cells of the B-lymphoid lineage or of hormone-responsive lineages mostly using transformed cell lines [6, 7, 9]. Recently CRISPR-Cas9 nuclease (Clustered Regularly Interspaced Short Palindromic Repeats (CRISPR)/CRISPR-associated systems) [10] was used to engineer human tumor-associated translocations [20]. To answer the question if locus proximity of *Pax3* and *Foxo1* in low-passage primary mouse myoblasts contributes to the frequency of *Pax3-Foxo1* fusion gene formation we used the CRISPR-Cas9 system to induce double strand DNA breaks (DSBs), which spurred by non-homologous end joining repair (NHEJ) produce chromosome translocations between these two loci. We used synthetic single-guide RNAs (sgRNA) to program Cas9 to induce DNA double-strand breaks (DSBs) in *Pax3* and *Foxo1* [21–23]. Unlike the human *PAX3/7* and *FOXO1* genes, mouse *Pax3/7* and *Foxo1* are in opposite orientation on their respective chromosomes (1, 4, and 3). Compared with human chromosome 13, *Foxo1* is part of an inverted 4.9Mb syntenic region on mouse chromosome 3. Although a recurrent complex inversion/translocation event involving the oppositely oriented *ETV6* and *c-ABL* genes in humans gives rise to the *ETV6-ABL* fusion gene in some myeloid and lymphoid malignancies, the frequency of this event is extremely low [24]. Therefore, to successfully generate a CRISPR-Cas9-mediated *Pax3-Foxo1* fusion gene we used chromosomal engineering via Cre recombinase-mediated genetic alterations to create a mouse in which the Foxo1 containing 4.9 Mb syntenic region is inverted (Foxo1-inv^+/+^ mice). Previously, Cre recombinase-mediated inversions of large fragments of chromosomes have been used to create balanced chromosomes [25–29].

We show that myoblasts isolated from fore and hind limb keep their Pax3-expressing identity and co-localization of *Pax3* and *Foxo1* loci strongly correlates with the level of Pax3 expression and generation of a CRISPR-Cas9 induced t(1;3), which is more frequent in fore limb myoblasts. Our Foxo1inv^+/+^ mice will be a valuable tool for studying mechanisms underlying the initial stages of the A-RMS implicated chromosome translocations resulting in development of better animal models for this pediatric cancer and other human diseases caused by chromosome translocations.

## Results

### Expression of *Pax3* in primary myoblasts correlates with co-localization of the *Pax3* and *Foxo1* loci

**Figure 1:**
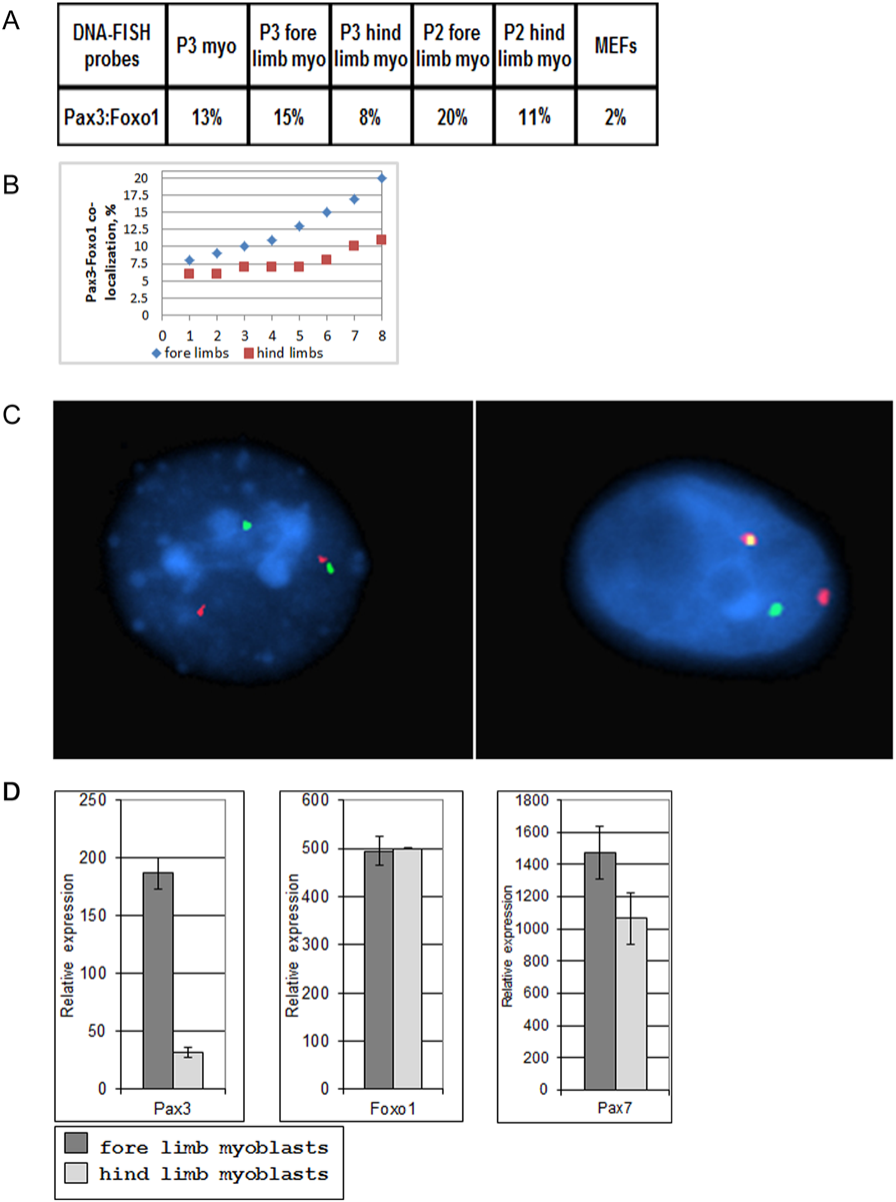
Co-localization of *Pax3* and *Foxo1* in primary mouse myoblasts. A. Percentages of *Pax3* and *Foxo1* co-localization in interphase nuclei of primary mouse myoblasts and MEFs as determined by FISH analysis using *Pax3* and *Foxo1* BAC probes. Myo = myoblasts; MEFs = mouse embryo fibroblasts. P2 and P3 represent the second or third passage of the myoblasts in culture, B. Compilation of eight independent *Pax3* and *Foxo1* co-localization experiments in interphase nuclei of primary fore limb and hind limb mouse myoblasts as determined by FISH analysis using *Pax3* and *Foxo1* BAC probes. Experiments1, 3, 5, 6, 7 and 8 were done with normal myoblasts, while experiments 2 and 4 were performed with Foxo1-inv+/+ myoblasts. This compilation includes the two fore limb and hind limb co-localization experiments shown in A.C. Confocal FISH micrographs showing co-localization of *Pax3* (shown in green) and *Foxo1* (shown in red) genes in interphase nuclei of mouse primary myoblasts. D. Q-RT-PCR analysis of *Pax3*, *Foxo1*, *Pax7* expression in primary mouse fore limb and hind limb myoblasts.

Since close physical proximity of translocation partners might facilitate chromosome translocation, we determined if *Pax3* and *Foxo1—*the translocation partners in A-RMS—are co-localized in actively proliferating low-passage primary mouse myoblasts. DNA-FISH analyses of the *Pax3* and *Foxo1* loci in interphase nuclei of primary limb myoblasts of newborn pups, after one week in culture showed 13% co-localization, which was significantly higher than in similarly cultured MEFs (2%, the background of this method; Fig. 1A). We hypothesized that co-localization of *Pax3* and *Foxo1* loci in myoblasts reflects the percentage of Pax3^+^ cells in the original newborn muscles. To test this hypothesis we isolated myoblasts from hind and fore limbs of newborn pups and compared the frequency of co-localization of *Pax3* and *Foxo1* loci in proliferating myoblasts from these two sources. It was shown previously with a *Pax3* knock-in reporter gene that many more satellite cells in fore limb muscle express *Pax3* than in hind limb muscle [19]. In accordance, the percentage of co-localized *Pax3* and *Foxo1* loci was notably higher in fore limb than in hind limb myoblasts in 8 independent experiments (Fig. 1A–C). In addition, Q-RT-PCR of RNA from these myoblasts confirmed that expression of *Pax3* was six-fold higher in fore limb myoblasts (Fig. 1D). These results are in agreement with the published observation that satellite cells maintain their Pax3^+^ identity upon activation *in vitro*. Expression of other genes such as *Foxo1* and *Pax7* was similar in the two types of myoblasts (Fig. 1D). The results for *Pax3* expression were reproducible given that a number of independent experiments produced similar data (S1 Fig.). Because diaphragm was shown to contain the highest number of Pax3^+^ myoblasts [19], we compared by FISH the co-localization of the *Pax3* and *Foxo1* loci in myoblasts isolated from fore limb, hind limb, and diaphragm of the same adult mouse. Indeed, diaphragm myoblasts showed a higher co-localization of the two loci (20%) than fore limb (11%) or hind limb (9%) myoblasts.

### Chromosome engineered Foxo1-inv^+/+^ mice

**Figure 2:**
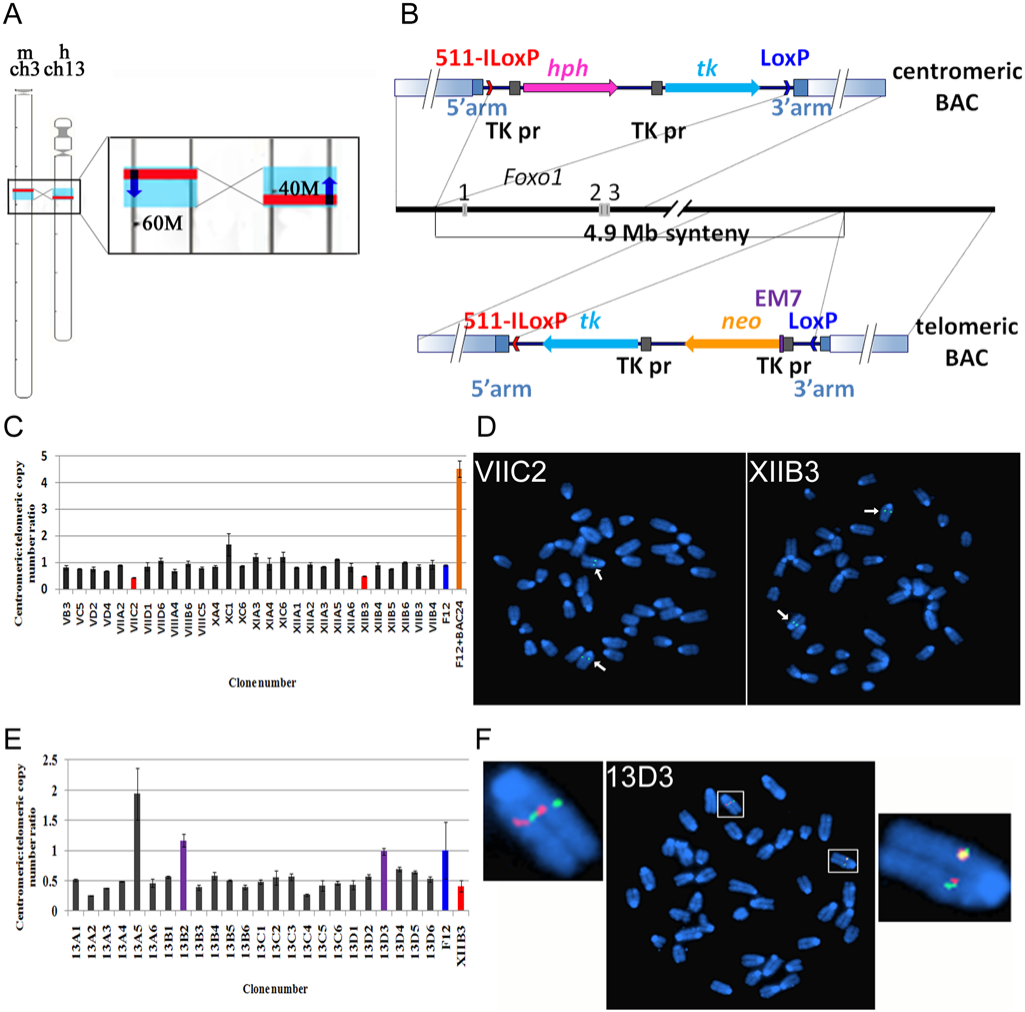
Strategy to invert a 4.9Mb region on mouse chromosome 3-containing *Foxo1*. A. Left, schematic representation of mouse chromosome 3 and human chromosome 13. Red lines indicate the relative positions of the *Foxo1* and *FOXO1* genes, blue boxes represent the syntenic regions. Right, enlargement of the chromosome areas boxed on the left showing the position of *Foxo1* and *FOXO1* (red rectangles), the position of the respective 4.9Mb syntenic regions (blue rectangles) and the transcriptional direction of the *Foxo1* and *FOXO1* genes (dark blue arrows). Numbers (60M, 40M) indicate the distance in Mb from the centromere. B. Scheme of two consecutive rounds of targeting in mouse ES cells of the centromeric (top) and telomeric (bottom) borders of the 4.9 Mb syntenic region. 511-ILoxP (red arrowhead) and LoxP (blue arrowhead)—two incompatible loxP sites, with arrowheads indicating their relative orientation; hph—Hygromycin-B resistance gene; neo—Neomycin resistance gene; tk—HSV1-thymidine kinase gene; TK pr—HSV1-thymidine kinase promoter; EM7—prokaryotic promoter; solid blue rectangles indicate homology arms used for recombineering in BACs; gradient blue rectangles indicate homology arms used for targeting in ES cells. C. Histogram representing the ‘loss-of-native allele assay’ to identify ES cell clones carrying a homologously targeted centromeric border of the 4.9 Mb syntenic region. Vertical bars of individual clones represent the copy number ratio of the native loci of the centromeric and telomeric borders of the 4.9 Mb synteny region. Only clones VIIC2 and XIIB3 exhibit a 1:2 signal ratio, indicating loss of one of the centromeric borders in these clones. F12 indicates non targeted F12 ES cell DNA; F12+BAC indicates wild type F12 ES cell DNA spiked with RP24–391O12 BAC DNA. D. FISH analysis of the VIIC2 and XIIB3 clones using the centromeric RP24–391O12 BAC probe. Arrows indicate two hybridization signals on chromosome 3. Because both clones are hygromycin resistant it indicates that they carry a correctly targeted centromeric border of the 4.9Mb syntenic region. E. Histogram representing the ‘loss-of-native allele’ assay to identify XIIB3 ES cell clones carrying a homologously targeted telomeric border of the 4.9 Mb syntenic region. Vertical bars of individual clones represent the copy number ratio of the native loci of the centromeric and telomeric borders of the 4.9 Mb synteny region. Clones 13B2 and 13D3 show a restored 1:1 signal ratio, indicating loss of one of the telomeric borders. F12 indicates non-targeted F12 ES cell DNA, XIIB3 indicates DNA of the clone with a targeted centromeric border. F. FISH analysis of 13D3 ES cell metaphase chromosomes using the RP24–391O12 (green = centromeric border) and RP23–422I13 (red = telomeric border) BAC probes. There are two green and two red hybridization signals on chromosome 3. Because clone 13D3 is neomycin resistant it carries a correctly targeted telomeric border of the syntenic region.

The mouse *Foxo1* gene is located on chromosome 3 in a 4.9 Mb DNA fragment (ch3:52,059,615–56,995,963) that is syntenic with human chromosome 13 (ch13:41,254,213–34,463,185) but positioned in the opposite orientation (Fig. 2A). This places *Foxo1* in the mouse in a reverse transcriptional direction with respect to that of the *Pax3* or *Pax7* genes. To engineer a mouse capable of acquiring productive *Pax3/7-Foxo1* fusion genes via a simple balanced t(1;3) or t(4;3), we performed two consecutive rounds of ES cell targeting in which we introduced two pairs of non-compatible *LoxP* sites at either border of this syntenic region with the goal to create a Cre-recombinase mediated permanent inversion of the 4.9Mb DNA fragment (S2 Fig.). Without inversion there would only be two ways to produce a productive fusion: 1) Via a translocation in which the resulting chromosomes would carry a double centromere and no centromere, respectively, an option likely to be non-viable in primary myoblasts and 2) Via a complex inversion/translocation event as described for the human *ETV6-ABL* fusion gene [24], a rare event, which likely would reduce the frequency of fusion gene formation below detectable levels.

The centromeric border of the mouse/human syntenic region is located 15 kb upstream of the *Foxo1* start codon (Fig. 2B). To precisely target this border in ES cells we used recombineering in *E. coli* [30] to modify the RP24–391O12 BAC (bacterial artificial chromosome) clone, so that it carries non-compatible mutant *511-ILoxP* and *wtLoxP* sites [31] flanking the *hph* (hygromycin B resistance) and *tk* (HSV1-thymidine kinase) selectable marker genes (Figs. 2B, S2). The precise targeting of the border of the syntenic region minimizes the chance of disturbing any potentially important regulatory sequences that might affect *Foxo1* expression (Fig. 2B, top). We targeted ES cells with linearized RP24–391O12-LoxP-hygro-TK BAC DNA and counter selected hygromycin B resistant clones carrying random integrations by screening for the presence of vector sequences remaining on either side of the insert. Colonies containing such vector segments were discarded [32]. The remaining clones were subjected to the ‘loss-of-native-allele’ assay using real-time quantitative PCR [33]. For copy number control of stably integrated target DNA we used the telomeric border of the syntenic region as a reference. In total 273 clones were analyzed, two of which contained a single copy of the wild type locus (Fig. 2C). These clones were submitted to FISH analysis and karyotyping which confirmed the presence of only two native signals on chromosome 3 when hybridized with a wild type BAC RP24–391O12 probe (Fig. 2D). For consecutive targeting of the telomeric border of the syntenic region we selected clone XIIB3, which had a 100% normal diploid karyotype.

For targeting of the telomeric border of the syntenic region we followed the same strategy and engineered a BAC clone carrying the 511-ILoxp-Neo-TK-wtLoxP cassette inserted at the precise syntenic border (Fig. 2B, bottom). The recombinant RP23–422I13-LoxP-Neo-TK BAC was linearized in such a way that only very short vector fragments remained at either side of the insert. After targeting in ES cells, analysis with the ‘loss-of-native-allele’ assay of 48 clones proved sufficient to obtain the desired recombinant. Two clones carrying a single copy of the wild type telomeric locus (Fig. 2E) were analyzed by FISH using the RP24–391O12 and RP23–422I13 BAC probes. One of them (13D3) showed two native signals on chromosome 3 with either BAC probe (Fig. 2F). This clone had a 90% normal diploid karyotype and we next determined if it carried cis- or trans-targeted borders of the 4.9 Mb syntenic region.

**Figure 3:**
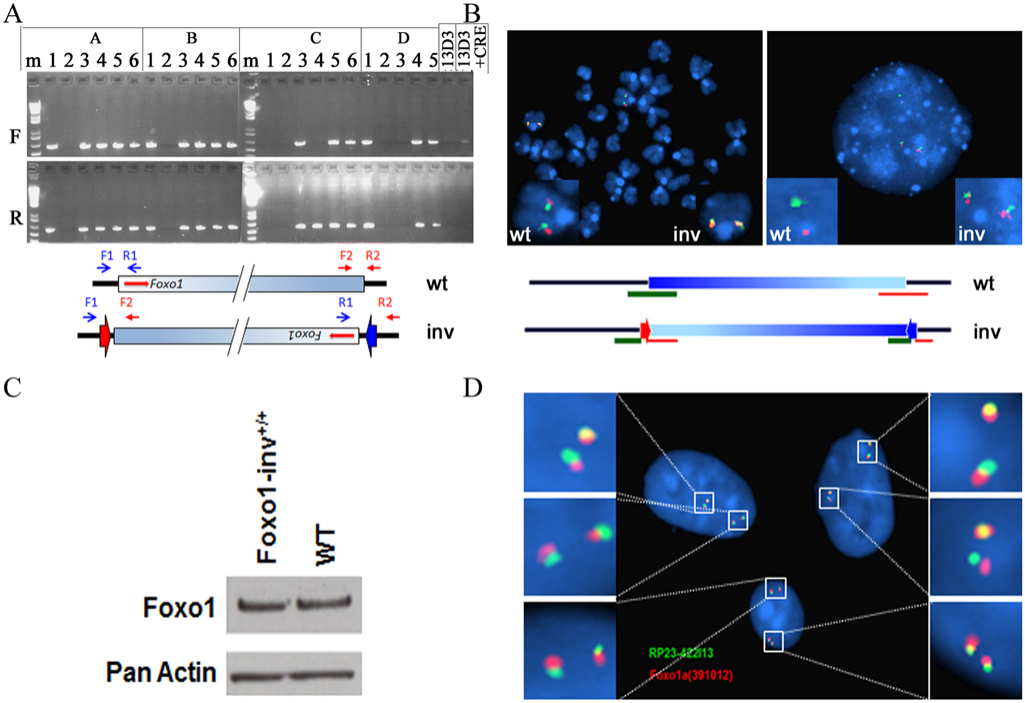
Analyses of inversion of the 4.9Mb syntenic region in double targeted ES cells. A. PCR analyses of CRE-transfected, FIAU selected ES cell clones. F, PCR amplification of the 4.9Mb borders using forward primers (RP24-F1 (blue arrow) and RP23-F2 (red arrow)); R, PCR amplification of the 4.9Mb borders using reverse primers (RP24-R1 (blue arrow) and RP23-R2 (red arrow)). Presence of PCR fragments in both the F and R PCR amplifications indicates the presence of the inverted 4.9M syntenic region. The scheme below the photograph shows the relative positions of the PCR primers on the wild type (wt) and inverted (inv) chromosomes, respectively. Arrowheads indicate the position of the remaining incompatible LoxP sites. Shaded blue box represents the 4.9Mb syntenic region, red arrows indicate the position and transcriptional orientation of *Foxo1*. B. FISH analyses of metaphase and interphase chromosomes of one of the ES cell clones carrying the 4.9Mb inversion using the RP24–391O12 (green = centromeric border) and RP23–422I13 (red = telomeric border) BAC probes. The wild type chromosome 3 (wt) produced contiguous red and green hybridization signals, the chromosome 3 with the inversion (inv) shows split hybridization signals. Left—metaphase spread; right—interphase nucleus. Scheme below the micrographs shows the positions of the two BAC probes on the wild type (wt) and inverted (inv) chromosomes, respectively. C. Western blot of cell lysates from Foxo1-inv^+/+^ and wild type (WT) myoblasts probed with a Foxo1 antibody (Foxo1). Detection of actin serves as a loading control. D. Interphase nuclei of fibroblasts from a mouse homozygous for the 4.9 Mb syntenic region inversion (Foxo1-inv^+/+^) showing split signals after in situ hybridization with the RP23–422I13 (shown in green) and RP24–391O12 (shown in red) BAC probes.

To discriminate between these two possibilities we transiently transfected a Cre recombinase plasmid into the double-targeted 13D3 ES cells and DNA isolated from the pool of electroporated cells was analyzed by PCR using only forward or reverse primers from both targeted borders. Both PCRs produced bands indicating that the pool contained cells carrying the 4.9Mb inversion. The same pool of cells was counter selected with FIAU and 23 resistant clones were analyzed by PCR (Fig. 3A). Sixteen clones harbored the 4.9Mb inversion and two of these were selected, A6 and C5, which had a 100% and 93% normal karyotype, respectively. Inversion of the 4.9Mb region in these clones was subsequently confirmed by FISH analysis (Fig. 3B) using the RP24–391O12 and RP23–422I13 BAC probes. The chromosome containing the inversion showed split hybridization signals while the wild type chromosome produced contiguous signals with these probes. These ES cell clones were used to generate chimeric mice that transmitted the inversion of the Foxo1 syntenic region to heterozygous Foxo1-inv^+/-^ offspring.

Foxo1-inv^+/-^ mice were fertile and produced Foxo1-inv^+/+^ offspring at the expected Mendelian frequency. Foxo1-inv^+/+^ animals did not exhibit any obvious phenotypic abnormalities and showed normal fecundity and life span. Moreover, western blot analysis confirmed that Foxo1-inv^+/+^ primary myoblasts and wild type myoblasts expressed equal amounts of Foxo1 protein (Fig. 3C) and co-localization of the *Pax3* and *Foxo1* loci was equal in Foxo1-inv^+/+^ and wild type myoblasts (8%, S5 Fig.). Finally, DNA-FISH analysis of Foxo1-inv^+/+^ fibroblasts with RP24–391O12 and RP23–422I13 BAC probes confirmed that both chromosomes 3 carried the 4.9Mb inversion (Fig. 3D).

### CRISPR-Cas9 induced t(1;3) reciprocal translocation

**Figure 4:**
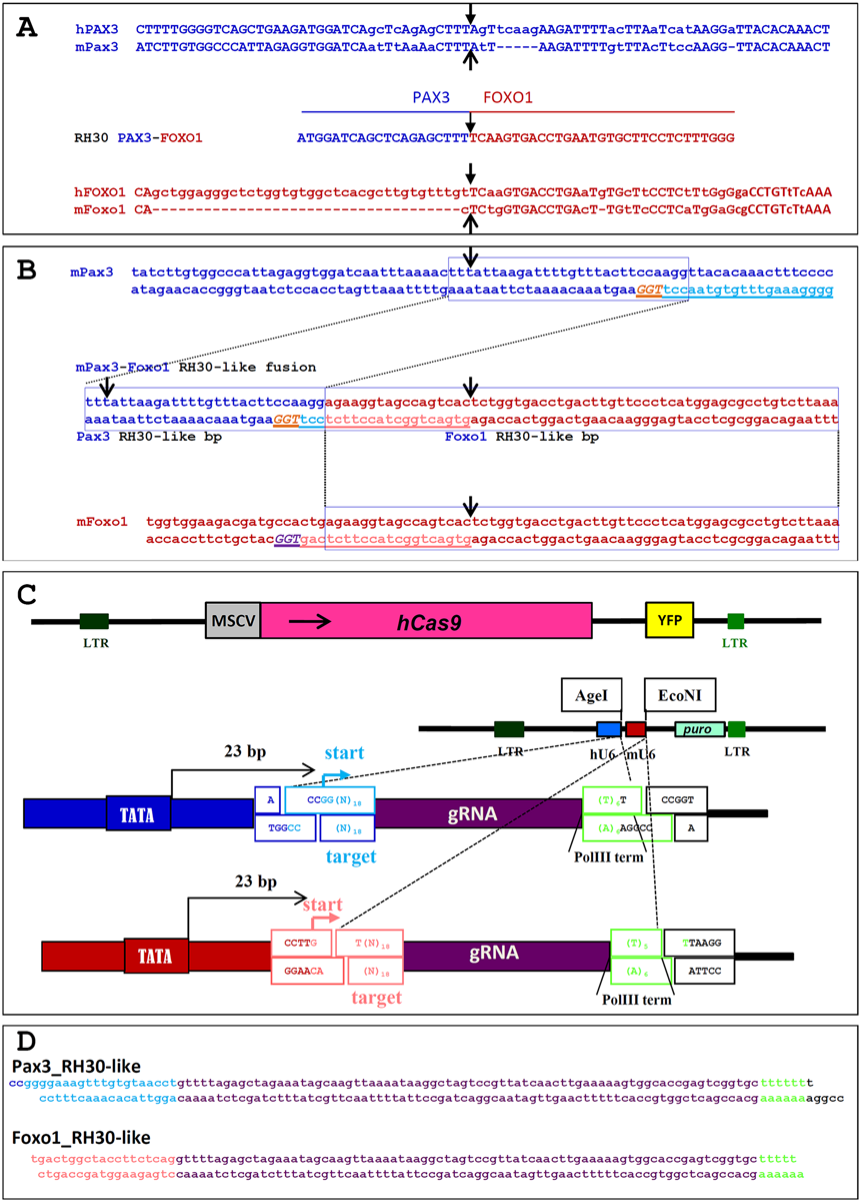
Sequences and vectors used for the generation of an RH30-like t(1;3) in Foxo1-inv^+/+^ mouse myoblasts. A. Top 2 lines show the sequence alignment of human *PAX3* intron 7 (hPAX3) with that of mouse *Pax3* intron 7 (mPax3) across the *PAX3* breakpoint (black arrow) in the A-RMS cell line RH30. Bottom 2 lines show the sequence alignment of human *FOXO1* intron 1 (hFOXO1) with that of mouse *Foxo1* intron 1 (mFoxo1) across the *FOXO1* breakpoint (black arrow) in RH30. The line in the middle shows the sequence of the t(2;13) breakpoint (black arrow) in RH30. B. Top 2 lines show the sequence of the mouse *Pax3* intron 7 across the RH30 breakpoint (black arrow). The targeting sequence of the sgRNA (light blue, underlined) and PAM sequence (orange, underlined) are indicated. hCas9 will generate a DSB 3 bp upstream of the PAM sequence. The bottom 2 lines show the sequence of the mouse *Foxo1* intron 1 across the RH30 breakpoint (black arrow). The targeting sequence of the sgRNA (pink, underlined) and PAM sequence (purple, underlined) are indicated. Cas9 will generate a DSB 3 bp upstream of the PAM sequence. The two lines in the middle show the sequence of the clean fusion between *Pax3* intron 7 and *Foxo1* intron 1 after Cas9 cleavage in Foxo1-inv^+/+^ myoblasts. Black arrows indicate the position of the RH30 *PAX3* and *FOXO1* breakpoints. The *Pax3* breakpoint is 25 bp downstream of that of the RH30 *PAX3* breakpoint, while the *Foxo1* breakpoint is 17 bp upstream of that of the RH30 *FOXO1* breakpoint. C. At the top is a schematic representation of the pCL20C-hCas9-IRES-YFP lentiviral vector. Below that is a schematic representation of the dual pCL20C-hU6-mU6-βact-puro sgRNA lentiviral vector containing the human (hU6) and mouse (mU6) U6 promoters driving the Pax3 and Foxo1 sgRNAs, respectively. Puro represents the SV40 early promoter driven puromycin resistance gene. Below the vector are magnifications of the borders of the hU6-sgRNA and mU6-sgRNA portions of the vector indicating the *AgeI* and *EcoNI* target RNA cloning sites and the DNA polymerase III sgRNA transcriptional start (start) and termination sites (PolIII term). LTR = long terminal repeat sequence, MSCV = MSCV-LTR, hCAS9 is human codon optimized Cas9, YFP = yellow fluorescent protein, D. Sequence of the RH30-like *Pax3* and *Foxo1* sgRNAs in the pCL20c-hU6-mU6-βact-puro vector.

Nuclear receptor-induced chromosomal proximity of *TMPRSS2* and *ERG* or *TMPRSS2* and *ETV1* promotes the occurrence of nonrandom ligation sites upon translocation between these partner genes, thereby generating unique breakpoint “hot spots” [6]. It is possible that translocations in A-RMS are non-random and occur predominantly at sites, coming in close proximity during co-regulated expression. We hypothesized that directing DSBs to sites in mouse *Pax3* and *Foxo1* homologous to those in *PAX3* and *FOXO1* in an ARMS cell line carrying a t(2;13) might increase the chance of generating a t(1;3) in proliferating Foxo1-inv^+/+^ myoblasts after Cas9 induced DSBs. We chose to mimic the breakpoints of the widely used ARMS cell line RH30 (S3 and S4 Figs.). Alignment of human and mouse *Pax3* and *Foxo1* sequences mapped the RH30-like breakpoints at positions 78105273 on mouse chromosome 1 and 52300558 on mouse chromosome 3 (Fig. 4A). We chose unique protospacer sequences followed by a 5’-GGT PAM as close as possible to the RH30-like breakpoints in both *Pax3* and *Foxo1* (Fig. 4B). Cas9 introduces DSB three nucleotides downstream of the two PAM sequences, which would result in DSBs between nucleotides 78105248 and 78105247 on chromosome 1 in intron 7 of *Pax3* and between nucleotides 52300541 and 52300542 (coordinates in the non-inverted sequence) on chromosome 3 in intron 1 of *Foxo1* (Fig. 4B).

For gene delivery to the myoblasts we cloned the human codon optimized Cas9 (hCas9) into the pCL20C [34] lentiviral vector downstream of the MSCV promoter and upstream of an IRES-YFP fluorescent marker (Fig. 4C). In order to express two different sgRNAs form a single vector we constructed a second pCL20C dual sgRNA vector in which the *Pax3*-specific sgRNA was driven by the human U6 promoter and the *Foxo1*-specific sgRNA by the mouse U6 promoter (Fig. 4C,D).

**Figure 5:**
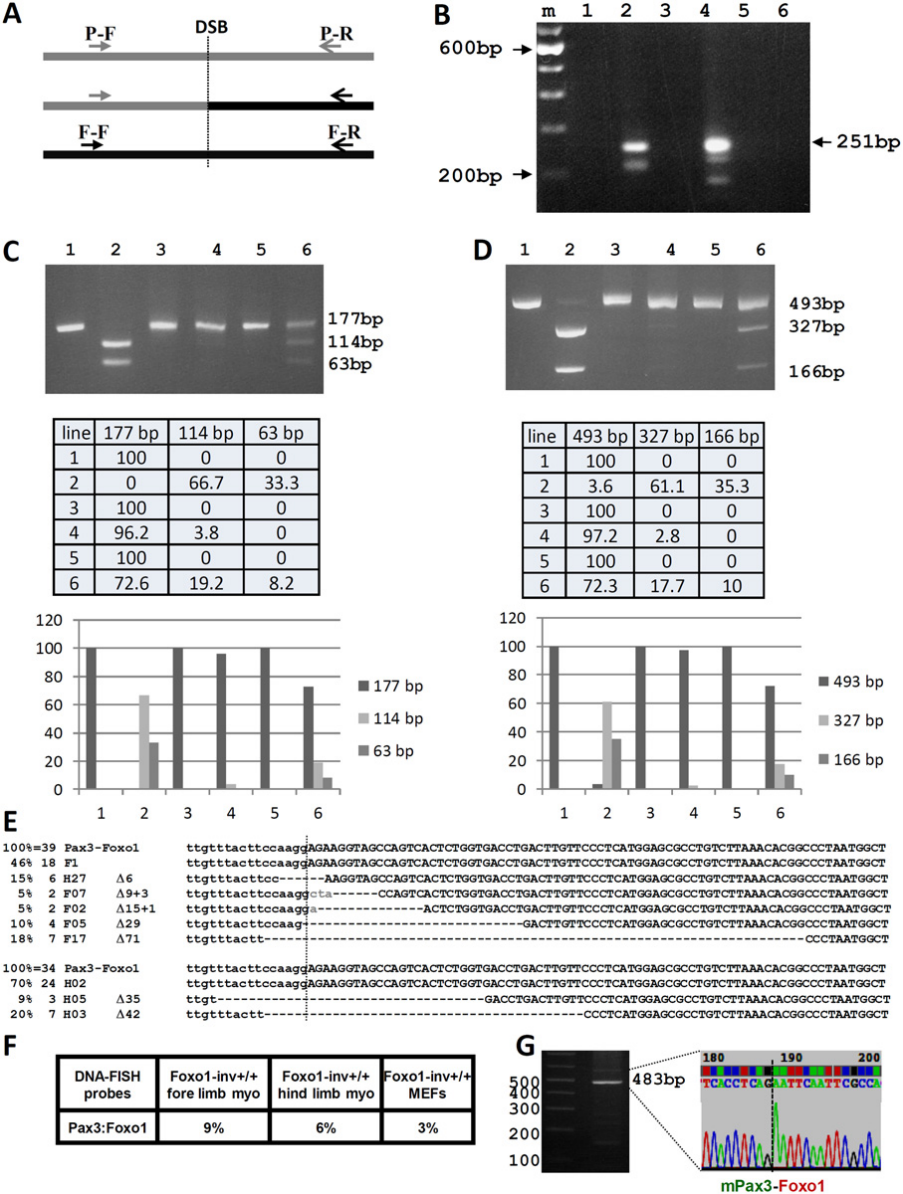
Analysis of the CRISPR-Cas9 induced t(1;3) in Foxo1-inv^+/+^ myoblasts. A. Schematic representation of the position of the *Pax3* forward (P-F) and reverse (P-R) PCR primers upstream and downstream of the *Pax3* breakpoint in intron 7 (DSB) and of the *Foxo1* forward (F-F) and reverse (F-R) PCR primers upstream and downstream of the *Foxo1* breakpoint in intron 1 (DSB). The *P-F and F-R primer pair amplify the Pax3-Foxo1 fusion*. B. Agarose gel showing the PCR products obtained using the P-F/F-R primer pair and DNA from Foxo1-inv^+/+^ hind limb DNA without (lane 1) and with (lane 2) CRISPR-Cas9 treatment, form Foxo1-inv^+/+^ fore limb DNA without (lane 3) and with (lane 4) CRISPR-Cas9 treatment, from wild type fore limb DNA with CRISPR-Cas9 treatment (lane 5), and from Foxo1-inv^+/+^ MEF DNA with CRISPR-Cas9 treatment (lane 6). M is 100 bp ladder molecular weight marker. The position of the expected size of the cleanly ligated *Pax3-Foxo1* fusion fragment is indicated on the right (251 bp). C. Agarose gel showing the *Pax3* PCR fragments containing the RH30-like Pax3 breakpoint from Foxo1-inv^+/+^ fore limb myoblast DNA not treated with CRISPR-Cas9 without (lane1) and with (lane 2) *MaeIII* digest, treated with CRISPR-Cas9 without (lane 3) and with (lane 4) *MaeIII* digest and Foxo1-inv^+/+^ MEF DNA treated with CRISPR-Cas9 without (lane 5) and with (lane 6) *MaeIII* digest. Expected fragment sizes are indicated on the right. Fully modified CRISPR-Cas9/NHEJ DSBs do not cut with *MaeIII*. Beneath the gel is a table with the relative fragment intensities as measured with a BIO-RAD ChemiDoc imaging system. Underneath the table is a histogram giving a graphic representation of the relative band intensities. D. Same analysis as in C but for the *Foxo1* fragment containing the RH30-like breakpoint digested with *DdeI*. The table underneath shows the relative fragment intensities as measured with a BIO-RAD ChemiDoc imaging system. Underneath the table is histogram giving a graphic representation of the relative band intensities. E. DNA sequences of 39 cloned *Pax3-Foxo1* PCR fragments from CRISPR-Cas9 treated fore limb (6 different) and of 34 cloned *Pax3-Foxo1* PCR fragments from CRISPR-Cas9 treated hind limb (3 different) myoblasts. F. Co-localization frequencies of *Pax3* and *Foxo1* in Foxo1-inv^+/+^ fore limb, hind limb, and MEFs used in B. G. Agarose gel and DNA sequence of a partial *Pax3-Foxo1* fusion cDNA fragment showing the correctly spliced *Pax3* exon7-*Foxo1* exon2 fusion after RT-PCR of RNA from CRISPR-Cas9 treated Foxo1-inv^+/+^ fore limb myoblasts.

We first determined that with our current batch of serum maximum co-localization of *Pax3* and *Foxo1* occurred at 7–8 days of culture after myoblast isolation. This time point synchronized with Cas9 and sgRNA expression should therefore maximize the probability of introducing DSB in closely positioned *Pax3* and *Foxo1* loci. Hence 24 hours after isolation we transduced primary fore and hind limb myoblasts of Foxo1-inv^+/+^ pups, Foxo1-inv^+/+^ MEFs and fore limb myoblasts from wild type mice with Cas9 lentivirus (Fig. 4C). After FACS sorting for YFP, cells were expanded and transduced with lentivirus expressing the RH30-like sgRNAs at day 7 after isolation and with an SV40 large T antigen expressing lentivirus at day 8. The latter was done to prevent senescence of the myoblasts during puromycin selection and allows subsequent expansion of the culture. To detect the *Pax3-Foxo1* fusion DNA fragments from Cas9/sgRNAs expressing myoblasts and MEFs we used the Pax3-RH30F (forward) and Foxo1-RH30R (reverse) primers for PCR analysis, which are positioned downstream and upstream of the putative Cas9-induced *Pax3* and *Foxo1* DSBs (Fig. 5A). PCR amplification of DNA from 10^4^ cells produced bands of 250 bp or shorter in Cas9/sgRNAs expressing hind limb and fore limb Foxo1-inv^+/+^ myoblast (Fig 5B, lanes 2 and 4). However, no product was detected upon PCR amplification of DNA from 10^4^ hind and fore limb Foxo1-inv^+/+^ myoblasts not treated with Cas9/sgRNAs (Fig. 5B, lanes 1 and 3) or from 10^4^ Cas9/sgRNAs expressing Foxo1-inv^+/+^ MEFs or wild type fore limb myoblasts (Fig. 5B, lanes 5 and 6). As a control we verified that the difference in translocation frequency between myoblasts and MEFs was not caused by differences in CRISPR-Cas9’s accessibility to chromatin, given that *Pax3* is not expressed in MEFs. The CRISPR-Cas9 breakpoint in *Pax3* falls within a *MaeIII* restriction endonuclease site and that in *Foxo1* within a *DdeI* site. Therefore we PCR amplified the Pax3 and Foxo1 fragments spanning the breakpoints and digested them with *MaeIII* or *DdeI*. This showed that 96% (*Pax3*) and 97% (*Foxo1*) of the PCR products of CRISPR-Cas9 treated myoblasts were resistant to *MaeIII* or *DdeI* digestion, whereas in CRISPR-Cas9 treated MEFs these numbers were 72% for both enzymes (Fig. 5C,D). Thus, there was no great difference in chromatin accessibility. Moreover, the *Pax3* and *Foxo1* chromatin in MEFs was equally accessible to CRISPR-Cas9, despite the fact that *Foxo1* is and *Pax3* is not expressed in these cells.

Cloning of the CRISPR-Cas9 induced fusion DNAs, followed by sequencing of 45 individual clones of each of the PCR products, produced 39 and 34 translocation breakpoint sequences from fore and hind limb myoblasts, respectively. This identified 6 different breakpoint sequences from fore limb and 3 different breakpoint sequences from the hind limb myoblasts. This represents the minimal number of translocation events per 10^4^ cells (Fig. 5E, top and bottom). Taking into account the percentage of locus co-localization (Fig. 5F) these numbers translate to a minimal translocation frequency of 1 in 150 in fore limb and 1 in 200 in hind limb myoblasts, respectively. The only sequence in common between the fusion fragments from these two types of myoblasts was the cleanly re-ligated fusion, without missing or added base pairs. The other 7 (5 from fore limb myoblast and 2 from hind limb myoblast) were all unique and carried NHEJ-mediated deletions varying from 6 to 71 bp. Superimposed on the deletion, two of the clones also contained randomly added base pairs. Notably, three additional breakpoint sequences obtained from an independent experiment (S5 Fig.) were different from the 7 shown in Fig. 5E and underline the mutation-prone repair of the NHEJ DNA-repair machinery during the translocation event. Together these results show excellent correlation between the frequency of translocation, co-localization, and expression of the *Pax3* and *Foxo1* loci in primary myoblasts. It was highest in fore limb myoblasts, lower in hind limb myoblasts and undetectable in MEFs. Although wild type myoblasts show the same frequency of locus co-localization as Foxo-inv+/+ myoblasts (S6 Fig.), the opposite orientation of Foxo1 prevented the formation of a productive *Pax3-Foxo1* fusion gene. Next we performed RT-PCR on equal amounts of total RNA from fore limb and hind limb myoblasts to detect the Pax3-Foxo1 fusion mRNA. In support of the higher frequency of chromosome translocation in fore limb myoblasts, we were able to RT-PCR amplify the *Pax3-Foxo1* cDNA from these myoblasts (Fig. 5G) but not from the hind limb myoblasts using an equal amount of input RNA (not shown). Sequence analysis of the cDNA confirmed the correctly spliced *Pax3* exon 7-*Foxo1* exon 2 fusion (Fig. 5G).

### FISH detection of the t(1;3) reciprocal translocation

**Figure 6:**
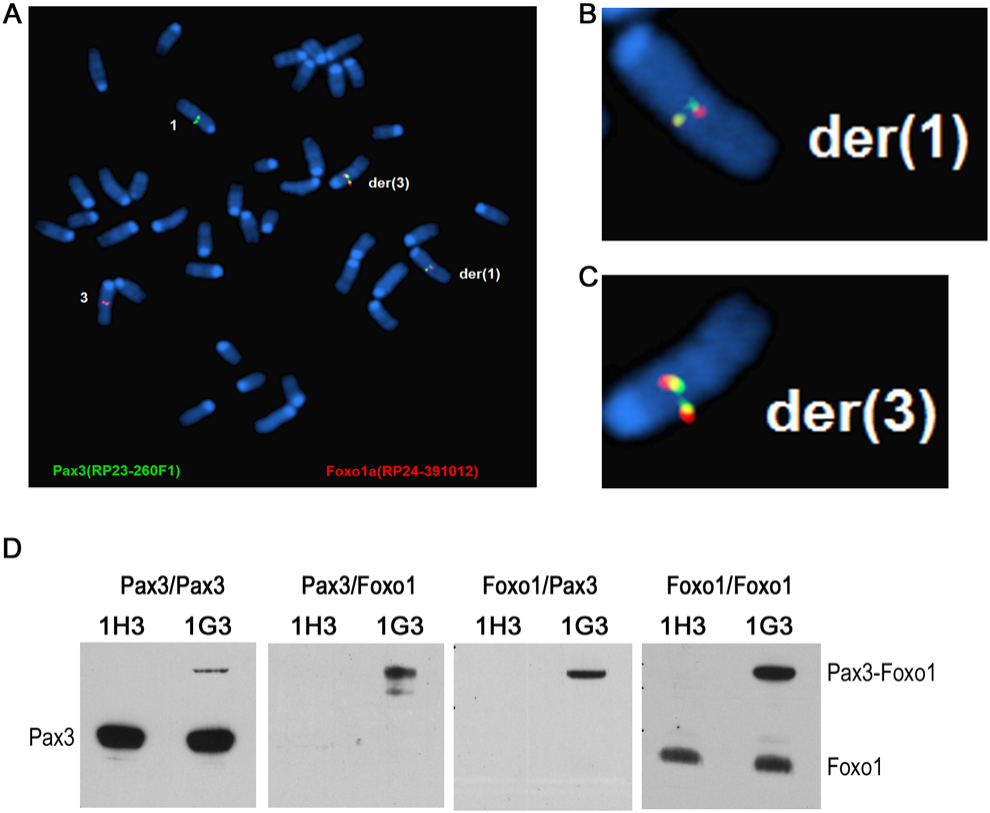
The reciprocal t(1;3) in myoblasts expresses Pax3-Foxo1 protein, which upregulates target gene expression. A-C. FISH analysis of an t(1;3)-enriched pool 1G3 myoblasts showing the reciprocal translocation. B and C show the magnified der(1) and der(3) chromosomes, respectively. D. Lysate of 1H3 (0%) and 1G3 [64% t(1;3)] myoblasts was immunoprecipitated with an anti-Pax3 antibody and immunoblotted with anti-Pax3 antibody (Pax3/Pax3) or anti-Foxo1 antibody (Pax3/Foxo1). The IP with anti-Foxo1 antibody was immunoblotted with anti-Pax3 antibody (Foxo1/Pax3) and an anti-Foxo1 antibody (Foxo1/Foxo1). The positions of the Pax3, Pax3-Foxo1 and Foxo1 bands are indicated.

To further characterize the t(1;3) we repeated the experiment in Foxo1-inv+/+/Ink4a-ARF-/- myoblasts. Due to loss of a functional p53 pathway Ink4a-ARF-/- myoblasts do not senesce during further experimental manipulation. Based on the *Pax3* and *Foxo1* co-localization data at the time of induction of the t(1;3) (11% in fore limb myoblasts and 7% in hind limb myoblasts) we assumed that the frequency of translocation events in these myoblasts should not be lower than in the myoblasts used in Fig. 5, i.e. at least 6 independent translocation events per 10^4^ fore limb myoblasts. This frequency is too low for further molecular and functional analyses. To enrich the cell pool for the t(1;3) carrying cells, we evenly distributed 10^4^ cells between the wells of three 96-well plates (on average 30 cells per well). PCR analyses of the DNA of 95 wells from the first plate identified 3 potentially t(1;3)-enriched cell pools (S7 Fig.). Pool 1E10 was lost during the freeze-thawing cycle but FISH analyses detected the reciprocal t(1;3) in 64% of pool 1G3 metaphase cells (Fig. 6A–C) and in 4% of pool 1D10 metaphase cells. Both the derivative chromosomes 1 and 3 were detected in all t(1;3) positive cells, confirming that the translocation was reciprocal.

### Functional analyses of cells harboring the reciprocal t(1;3)

To determine if the t(1;3) resulted in expression of Pax3-Foxo1 protein we immunoprecipitated three cell lysates each of the t(1;3)-negative (1H3) and t(1;3)-positive (1G3) pools with either an anti-Pax3 or an anti-Foxo1 antibody. The Pax3 IPs were then immunoblotted with the anti-Pax3 antibody, showing the Pax3 and Pax3-Foxo1 bands (Fig. 6D, Pax3/Pax3 panel), or with anti-Foxo1 antibody showing only the Pax3-Foxo1 band (Fig. 6D, Pax3/Foxo1 panel). Similarly, immunoblotting the Foxo1 IPs with anti-Pax3 antibody again showed the Pax3-Foxo1 fusion protein (Fig. 6D, Foxo1/Pax3 panel) while immunoblotting with the Foxo1 antibody showed both Foxo1 and the fusion protein (Fig. 6D, Foxo1/Foxo1 panel). This confirmed that the engineered t(1;3) expressed the fusion protein, which allowed us to assess if it affected the expression of Pax3-Foxo1’s transcriptional targets. We performed RNA-seq analysis comparing the mapped sequence reads of presumed PAX3-FOXO1 target genes [2] in the 1G3 pool (64% Pax3-Foxo1 positive) with those in the 1H3 pool (Pax3-Foxo1 negative) (S1 Table). This showed that roughly half the targets of PAX3-FOXO1 were correctly up or down regulated in the 1G3 pool. The same comparison with a PAX3-FOXO1 expression signature obtained with the ectopic PAX3-FOXO1 expressing ERMS cell line RD [35], also showed coincident regulation of half the targets (S2 Table), suggesting that the t(1;3) generated fusion protein is active.

## Discussion

For the precise modeling of human recurrent chromosome translocations and their impact on disease development in mice, reenactment of the actual translocation would be the closest possible recapitulation of the sequence of events in humans. Until now such reenactment was a daunting task as the translocation would require introduction of *LoxP* [36, 37] or *Frt* recombination sites into both translocation partners via homologous recombination in ES cells, followed by expression of Cre or Flp recombinase to create DSBs that would mediate the translocation. As shown by others [20] and here, the availability of the CRISPR-Cas9 system has paved the way to implementing this approach without such major technical or time investment. Given the high homology between mouse and human genes and their regulatory sequences, this approach is likely to include all sequences that are important for the precise regulation of the mouse fusion gene as it occurs in humans. The first and only published model for ARMS [38] in which expression of a conditional *Pax3-Foxo1* knock-in fusion oncogene is induced by a *Myf6* driven Cre had a low incidence and long latency of tumor development, requiring the presence of two *Pax3-Foxo1* alleles on a Trp53-null or Ink4a/Arf-null background. One reason for this might be that the level of expression of the fusion oncogene in this KI model is inadequate for shorter latency tumor development. An argument against this possibility is that a high level of PAX3-FOXO1 expression induces cell death [39], most likely due to transcriptional activation of the *Pax3-Foxo1* pro-apoptotic target gene *Noxa1* [40]. Unlike other studies [41, 42], the KI *Pax3-Foxo1* gene contained some *Foxo1* genomic sequences that allowed expression of the fusion gene in adult mice, but despite their presence the construct might lack sequences that mediate human-like regulation of fusion gene expression, which in turn might be crucial for efficient tumor development.

In agreement with published data [19] we established that co-localization of *Pax3* and *Foxo1* in our culture system was higher in forelimb than in hind limb myoblasts, which coincided with higher Pax3 expression in forelimbs. Due to experimental variability the percentage of co-localization of the two loci varied in 8 independent experiments, but co-localization in the fore limbs was always higher than in the hind limbs. Therefore our myoblast model represents a graded system to determine if these features contributed to the frequency of chromosome translocation in low passage primary myoblasts upon introduction of targeted DSBs. To perform these experiments and to eventually develop a precise mouse model of ARMS, the transcriptional orientation of *Foxo1* on chromosome 3 needed to be inverted. We followed the Cre-dependent one-way inversion of a DNA fragment in mice as was previously demonstrated by Schnütgen and colleagues [43]. To avoid disturbing the transcriptional regulation of the inverted *Foxo1*, we decided to invert the mouse/human 4.9 Mb syntenic region encompassing *Foxo1*, rather than the gene itself. Although the centromeric border of this region is only 15 kb upstream of *Foxo1*, we reasoned that all important *Foxo1* regulatory sequences should be contained within this region otherwise it would not be syntenic with human *FOXO1* on chromosome 13q14.1. Although we did not analyze the detailed expression of *Foxo1* in Foxo1-inv^+/+^ mice during pre- and postnatal life, the animals did not show any obvious phenotypic abnormalities. In addition, they had a normal lifespan, normal fecundity, and the level of Foxo1 protein expression and co-localization of the *Pax3* and *Foxo1* loci in myoblasts were identical to those of wild type mice. Together these observations made the Foxo1-inv^+/+^ myoblasts suitable for our translocation experiments.

To determine if the level of co-localization of *Pax3* and *Foxo1* in primary myoblasts affected the frequency of chromosome translocation between these loci upon induction of targeted DSB, we transduced the cells with Cas9 and dual sgRNA expressing lentiviruses. Combining the three genes into a single lentiviral vector failed to produce viral particles. We targeted the CRISPR-Cas9 DSBs to sequences in *Pax3* and *Foxo1* that mediated the t(2;13) in the A-RMS cell line Rh30. Both breakpoints are present in sequences conserved between the mouse and human genes, suggesting that they occurred in non-redundant sequences that might bind factors with a role in expression regulation of both genes. Currently we do not know if this affects the frequency of translocation, which is a possibility that can be tested in future by choosing sgRNAs targeting non-conserved sequences within the target *Pax3* and *Foxo1* introns. We found excellent positive correlation between the frequency of the t(1;3) and the percentage of locus co-localization using FISH analysis. This also correlated with the level of Pax3 expression, which is much higher in fore limb than hind limb myoblasts and absent in MEFs, while Foxo1 expression is ubiquitous. Given that the frequency of CRISPR-Cas9 induced DSBs in *Pax3* and *Foxo1* is comparable in myoblasts and MEFs, it is the proximity of the loci in these primary cells that facilitates trans-chromosomal ligation producing the two expected derivative chromosomes during NHEJ DNA repair. The derivative chromosome 3 produced correctly spliced *Pax3-Foxo1* mRNA, encoding active Pax3-Foxo1 protein that up/down-regulated expression of approximately half the presumed PAX3-FOXO1 targets in the 64% *Pax3-Foxo1*-positive cell pool (S1 Table). The genes compiled in this table are differentially expressed in ARMS versus ERMS tumors or have been identified by forced expression of PAX3-FOXO1 in different cell lines, including NIH3T3 cells, MEFs, SAOS2 cells and C2C12 cells ([2] and references therein). Because the cell background affects the range of PAX3-FOXO1 target gene expression [44], none of the published scenarios reflect expression of Pax3-Foxo1 in primary p16/Arf^-/-^ mouse myoblasts. Possibly this is the reason for the 45% match of reported PAX3-FOXO1 up or down regulated genes. Comparison with genes up or down regulated in the ERMS cell line RD transduced with *PAX3-FOXO1* retrovirus [35] showed 52% coincident regulation (S2 Table). Clearly, the t(1;3) generated Pax3-Foxo1 protein in mouse myoblasts is active and changes the expression of target genes in an ARMS-like manner.

One would expect that the frequency of translocation in myoblast that show co-localization of the two translocation partners would be the same irrespective of the source of myoblasts. We found a frequency of 1/150 and 1/200 in fore and hind limb myoblasts, respectively, which we believe does not represent a difference given the uncertainty of how many translocation events actually took place (we can only count those that give distinguishable fusion products). Our results in mouse myoblasts suggest that human myoblasts can be a cell of origin for the *PAX3-FOXO1* translocation as they would provide a favorable environment for the translocation to occur, i.e. expression of both genes and spatial co-localization. It is curious that A-RMS is more frequent in the lower than in the higher extremities in humans, as reported by Neville and co-workers [45]. This apparent inconsistency with our mouse data might be explained by the possibility that humans may not have a difference in the distribution of PAX3 expression in the upper and lower extremities. In addition, the muscle mass and presumably the number of satellite cells in the lower extremities in humans is much higher than in the upper extremities, hence increasing the number of translocation-competent cells and frequency of translocation.

By using CRISPR-Cas9 nuclease we showed that targeted chromosome translocations could be induced with high efficiency. Unlike other approaches that have relied on induction of chromosome translocation using γ−irradiation, DSB-inducing chemicals, or the lymphoid cell-specific gene rearrangement machinery, CRISPR-Cas9 can be employed in any cell type. Due to its specificity the system is suitable for use *in vivo* in cell culture or in mice. Application of this system will greatly facilitate the development of mouse models that precisely recapitulate chromosome translocation-induced human diseases.

## Materials and Methods

### Strains of *E. coli*, BAC clones, PCR Primers and oligonucleotides

A complete list of *E. coli* strains used for this work can be found in S1 Protocol.

BAC clones RP24–391O12 (centromeric border of the 4.9 Mb syntenic region) and RP23–422I13 (telomeric border of the 4.9 Mb syntenic region) were purchased from the BACPAC Resource Center (BPRC), Children’s Hospital Oakland Research Institute in Oakland, California, USA (http://bacpac.chori.org).

The complete list of PCR Primers and oligonucleotides can be found in S1 Protocol.

### Construction of plasmid and BAC-based targeting vectors

A modified pNeoTKLoxP was recombineered into BAC RP23–422I13 (telomeric border of the syntenic region). In pNeoTKLoxP we replaced the wild type (wt) LoxP site downstream of the TK gene with the 511-ILoxP sequence (annealed oligonucleotides TK-511-ILoxP and TK-511-ILoxP-C). Then, via recombineering, we introduced the EM7 promoter upstream of the Neo gene. We therefore transformed electrocompetent DY380 *Ecoli* cells, containing the wtLoxPNeoTK-511-IloxP plasmid with the TK-EM7-Neo fragment (ends of the annealed oligonucleotides TK-EM7 and EM7-NeoC had been filled-in with Klenow DNA polymerase (Invitrogen) following the manufacturer’s protocol). For recombineering we followed the protocol posted on the Frederick National Laboratory for Cancer Research web site: http://ncifrederick.cancer.gov/research/brb/protocol/Protocol1_DY380.pdf. A short 5’-arm (annealed phosphorylated oligonucleotides 5-tel-s and 5-tel-s-C) was cloned downstream of 511-IloxP and a short 3’-arm was cloned upstream of wtLoxP (annealed phosphorylated oligonucleotides 3-tel and 3-tel-C).

A modified pBSLoxPTKhygro plasmid (kind gift from Drs. M. Roussel and F. Zindy, SJCRH) was recombineered into BAC RP24–391O12 (centromeric border of the syntenic region). In this construct we inserted a 511-ILoxP sequence upstream of the TK-promoter-Neo sequence. Since the activity of TK promoter in prokaryotic cells was sufficient to ensure Hygromycin B resistance, we did not introduce the bacterial EM7 promoter in this construct. A short 5’-arm (annealed phosphorylated oligonucleotides 5-cent-s and 5-cent-s-C) was cloned upstream of the 511-IloxP site and a short 3’-arm was cloned downstream of wtLoxP (annealed phosphorylated oligonucleotides 3-cent and 3-cent-C).

The pCL20c-MSCV-IRES-YFP vector backbone was generated by replacing GFP of pCL20c-MSCV-GFP [46] with I-YFP from MSCV-I-YFP [38]. hCas9 [23] was then cloned downstream of MSCV into pCL20c-MSCV-IRES-YFP. The mU6 fragment was generated by PCR using pSicoR-GFP (Addgene, Cambridge, MA, USA) and cloned downstream of hU6 in pLKO.1 (Addgene, Cambridge, MA, USA). The cassette containing the human and mouse U6 promoters (hU6 and mU6) followed by AgeI and EcoNI cloning sites was cloned upstream of the β-actin promoter of the modified pCL20c vector, containing the β-actin-puro cassette from pJ6.OMEGA.puro [47]. The spacer sequence of hU6 driven sgRNA starts with GG followed by 18 specific nucleotides from the target sequence, and mU6 driven sgRNA starts with GT followed by 18 specific nucleotides from the second target sequence (Fig. 4C). Synthetic ds-DNA fragments, coding Pax3_RH30 sgRNA and Foxo1_RH30 sgRNA were cloned into AgeI and EcoNI sites under control of hU6 and mU6 promoters of pCL20C-hU6-mU6-βact-puro, respectively (Fig. 4C,D).

pCL20C-MSCV-Luc2–2A-LgT was constructed by replacing IRES-YFP with a Luc2–2A-LgT cassette.

### Lentivirus production

Lentivirus was produced as described in [46].

### ES cell targeting, Cre-mediated inversion, and screening of ES cell clones

F12 (129SvJ-derived) embryonic stem (ES) cells were electroporated and selected for hygromycin B or G418 resistance using standard procedures. In short, 25–45 μg of linearized BAC DNA was electroporated into 2*10^7^ ES cells followed by selection with 100 μg/ml Hygromycin B or 200 μg/ml G418. RP24–391O12-LoxP-hygro-TK was linearized with PI-SceI (NEB) and RP23–422I13-LoxP-Neo-TK was linearized with NotI (NEB). Drug resistant clones were picked after 7–9 days of selection. DNA from these clones was used for PCR analysis.

Screening of homologously recombined ES cell clones was done by PCR and qPCR. The presence of vector arms remaining on either side of the insert was detected by PCR with primers pTARBAC1–3F and pTARBAC1–3R for 3’-located sequences and pTARBAC1–5F and RP24–5R for 5’-located sequences. The “loss-of-native allele” assay was performed as described in [33] with some minor modification. For quantitative (q)PCR we used SYBR®Green PCR Master Mix (Applied Biosystems). qPCR was performed with the RP24-F and RP24-R primers to determine the copy number of the centromeric locus and the RP23-F and RP23-R primers to determine the copy number of the telomeric locus. Ratios between the copy numbers of the two loci were determined either by a standard dilution curve or by the Δct method.

A double targeted ES cell clone was electroporated with a Cre-expressing plasmid (pMC-CRE) using the Amaxa™ Mouse ES Cell Nucleofector™ Kit (Lonza, Germany) according to the manufacturer’s protocol. After 5 days of selection with 0.2μM of fialuridine (FIAU) (a kind gift of Bristol Myers) ES cells were collected for DNA isolation as a pool or as single clones. Cre-mediated inversion was detected by standard PCR using the RP24-F/RP23-F2, and RP24-R/RP23-R2 primer pairs.

### Fluorescence *in situ* hybridization (FISH)

For FISH analyses of *Pax3* and *Foxo1* co-localization and detection of t(1;3) reciprocal translocation we used BAC probes RP23–260F1 and RP24–391O12. For FISH analyses of targeted ES cells and Foxo1-inv^+/+^ fibroblasts we used BAC probes RP24–391O12 (centromeric border of the syntenic region) and RP23–422I13 (telomeric border of the syntenic region). BAC probes were labeled with nick translation using either Green (RP23–260F1and RP24–391O12) or Red (RP23–422I13) dUTP (Abbott Molecular). Probes were hybridized to metaphase and/or interphase cells either separately or as a 1:1 mixture in hybridization solution (50% formamide, 10% dextran sulfate, and 2X SSC). Slides were washed in 2X Saline-Sodium Citrate (SSC) buffer containing 50% formamide at 37°C for 5 minutes. Cells were counterstained with DAPI and analyzed using a Nikon E80i fluorescence microscope (Nikon) with a 100× oil immersion objective. Successfully targeted clones showed 2 native signals for the centromeric or telomeric targeted regions. Inverted chromosomes 3 appeared as two linked pairs of red and green signals on interphase cells, each pair representing one end of the inverted chromosome segment. Normal chromosomes 3 appeared as a single loosely paired red and green signal. One hundred interphase nuclei were scored for the presence of co-localization of Pax3/Pax7 and Foxo1 signals. Only nuclei with discernible red and green signals were scored. Fifty metaphase cells from CRISPR-Cas nuclease treated myoblasts were scored for the presence of cells containing the reciprocal translocation between *Pax3* and *Foxo1a*.

### Identification of the translocation breakpoints in A-RMS

Experimental details are provided in S1 Protocol. Position of primers, used for LD-PCR, gel electrophoresis of LD-PCR products and sequences flanking the breakpoint in the Rh30 cell line are shown in S3 and S4 Figs.

### Detection of RH30-like translocation in primary myoblasts

Cas9 induced translocation was detected by PCR of chromosomal DNA from 10^4^ cells using Pax3-RH30F and Foxo1-RH30R primers.

### qRT-PCR and RT-PCR of myoblasts

For each qRT-PCR reaction we used RNA isolated from either 2.5×10^3^ (data in Fig. 1) or 1.6×10^3^ (data in S1 Fig.) cells. For each RT-PCR we used RNA isolated from 6.7×10^3^ cells. For RT we used SuperScript III First-Strand Synthesis SuperMix (Invitrogen) with an equimolar mix of Pax3R primer and random hexonucleotides and performed the reaction following the manufacturer’s protocol. For the qPCR step we used TaqMan Gene Expression Master Mix (Applied Biosystems). Ratios between gene expression in different cell lines were determined by a standard dilution curve.

### Immunoprecipitation and western-blot analyses

Myoblasts (5×10^6^) were lysed in 0.5 ml CHAPS lysis buffer (40 mM HEPES [pH 7.4], 1 mM EDTA 120 mM NaCl, 10 mM sodium pyrophosphate, 10 mM β-glycerophosphate, 0.3% CHAPS, 50 mM NaF, 1.5 mM NaVO, 1 mM PMSF, and 1 tablet of EDTA-free protease inhibitors [Roche] per 10 mL solution) and freeze-thawed 3 times, followed by centrifugation at 20,000 ×*g* for 10 min at 4°C. After adding 2 μg anti-Pax3 antibody [48] or anti-Foxo1 (C29H4) Rabbit mAb (Cell Signaling) Pax3, Foxo1 and Pax3-Foxo1 were immunoprecipitated overnight at 4°C. Immunoprecipitated material was bound onto 10 μl protein G-coated Dynabeads (Invitrogen) for 90 minutes at 4°C, which were captured using a DYNA-Mag-2 magnet (Invitrogen), washed 4 times with CHAPS buffer, and removed from the beads by heating to 70°C in 1.25xLDS loading buffer (Invitrogen) in CHAPS and separated on pre-cast 4%–12% bis-tris polyacrylamide gels. Western-blotting was performed using the same anti-Foxo1 and anti-Pax3 antibodies.

### Enrichment of t(1;3) harboring cells

To enrich for cells harboring the t(1;3), 10^4^ of the Cas9/sgRNAs expressing fore limb myoblasts were evenly distributed over three 96-well plates (on average 30 cells per well). DNA from each cell pool was isolated and analyzed for the presence of t(1;3) translocation using PCR.

### RNA sequence analysis

Libraries were generated from ~ 500 ng total RNA of the 1H3 (no Pax3-Foxo1) and 1G3 (64% Pax3-FOXO1) cell pools using the Illumina TruSeq Stranded mRNA Sample Preparation Kit. Libraries were sequenced on an Illumina HiSeq 2500 using paired-end 100 bp sequencing chemistry. Paired-end reads from RNA-seq were aligned to the following 4 database files using BWA (0.5.10) aligner: (1) the human GRCh37-lite reference sequence, (2) RefSeq, (3) a sequence file representing all possible combinations of non-sequential pairs in RefSeq exons, (4) AceView database flat file downloaded from UCSC representing transcripts constructed from human ESTs. The mapping results from (2) to (4) were aligned to human reference genome coordinates. In addition, they were aligned using STAR 2.3.0 to the human GRCh37-lite reference sequence without annotations. The final BAM file was constructed by selecting the best alignment among the five map events. We used HTSeq [49] to count the number of fragments that mapped to each gene (Gencode v 15), where each gene is considered as the union of all its exons. Then we normalized the count to FPKM (fragments per kilobase of exons per million fragments mapped) as the expression value of the gene. RNA-seq of both samples produced 55M reads each, with a 20X coverage of 43.561% of the exons in 1H3 and 43.992% of the exons in 1G3.

### Ethics statement

This study was performed in strict accordance with the recommendations in the Guide for the Care and Use of Laboratory Animals of the National Institutes of Health. The Institutional Animal Care and Use Committee (IACUC) of St. Jude Children’s Research Hospital (Animal protocol number 209–100171) approved the protocol.

## Supporting Information

S1 Fig Q-RT-PCR analysis of *Pax3*, *Foxo1*, *and Pax7* expression in primary mouse fore limb and hind limb myoblasts. Upper 3 panels: Q-RT-PCR of a second fore and hind limb myoblast isolation experiment showing expression of *Pax3*, *Foxo1* and *Pax7* in both types of myoblasts. Lower 3 panels Q-RT-PCR showing the difference in *Pax3* expression in fore and hind limb myoblasts in 3 additional, independent myoblast isolations.
(PPT)

S2 Fig CRE recombinase-induced irreversible inversion of a 4.9Mb region on mouse chromosome 3 in ES cells. 1°—Schematic representation of the mouse/human 4.9 Mb syntenic region (gradient blue rectangle) on wild type mouse chromosome 3. The red arrow inside the gradient blue rectangle indicates the position and transcriptional direction of *Foxo1*. The arrows above the rectangle indicate the positions of the blue F1 centromeric and the red F2 telomeric forward primers and the blue R1 centromeric and red R2 telomeric reverse primers. 2°—Mouse chromosome 3 after consecutive centromeric and telomeric targeting of the borders of the 4.9 Mb syntenic region. Arrows underneath 511-ILoxP and LoxP—two incompatible loxP sites, indicate their relative orientation; hph—Hygromycin B resistance gene; neo—G418/Neomycin resistance gene; tk—HSV1-thymidine kinase gene. 3°A—First step of Cre-induced inversion of the double-targeted 4.9 Mb region. If the LoxP sites are used, the inversion of the 4.9Mb fragment places one LoxP site, the *tk* genes, and the antibiotic resistance genes between head-to-tail oriented 511-ILoxP sites. 3°B—Alternative first step of Cre-induced inversion of the double-targeted 4.9 Mb region. If the 511-ILoxP sites are used, the inversion of the 4.9Mb fragment places one 511-ILoxP site, the *tk* genes, and the antibiotic resistance genes between tail-to-head oriented LoxP sites. 4°—Cre-induced recombination via the 511-ILoxP (3°A) or the LoxP (3°B) sites results in the irreversible inversion of 4.9M syntenic region and flips the transcriptional orientation of *Foxo1a*.
(PPT)

S3 Fig Localization of primers used in LD-PCR. The human sequence of *PAX3* intron 7 (A) and *FOXO1* intron 1 (B) was compared to the mouse sequence using the ECR Browser genome analysis tool to show the location of evolutionarily conserved regions within these loci. Sequence comparison was performed with an ECR window of 100bp with a minimum similarity of 70%. The position of the exons (blue boxes) is indicated as well as the 5’ to 3’ direction of the gene (blue arrows). Blue peaks correspond to coding exons, yellow peaks correspond to 5’ or 3’ untranslated regions, orange peaks correspond to intronic non-coding conserved sequences and green peaks correspond to repetitive sequences. Primer positions are indicated (black arrows) with their names shown above. The *PAX3* primers *PAXLD1–7* are designated pP1–7. Primers *PAXLD1–6* were designed 3kb apart from each other to span the *PAX3* intron 7. Primer *PAXLD7* is located immediately upstream of *PAX3* exon 7. The *FOXO1* primers *FOXLD1–12* are designated pF1–12. Primers *FOXLD1–11* are located 10kb apart from each other. Additional primers were designed between primers *FOXLD4–11* to reduce the primer interval to 5kb. Primer *FOXLD12* is located immediately downstream of exon 2.
(TIF)

S4 Fig Amplification of the translocation breakpoint of the derivative chromosome 13 of the ARMS cell line Rh30. (A) Gel electrophoresis of LD-PCR products obtained in the amplification across the breakpoint in the Rh30 cell line. Lanes 1–3: reverse primer *FOXLD6* in combination with *PAXLD2*, *PAXLD3* and *PAXLD4* generated fragments of 5.8kb, 8.8kb and 11.8kb, respectively. Lane 4: primers *FOXLDwt* and *FOXLD8* resulting in a 10.1kb long fragment (positive control). Lane 5: 1kb DNA ladder (Invitrogen). (B) Sequences flanking the breakpoint in the Rh30 cell line showing the seamless transition between chromosomes 2 and 13.
(TIF)

S5 Fig Additional *Pax3-Foxo1* fusion sequences from an independent CRISPR-Cas9 translocation experiment. Three additional fore limb myoblast *Pax3-Foxo1* fusion sequences are shown below the predicted *Pax3-Foxo1* fusion sequence. These were generated in an independent experiment producing sequences that are distinct from those depicted in Fig. 5E. Nucleotides in lower case represent Pax3 sequences, capitals represent Foxo1 sequences. Nucleotides in red lower case have been added randomly via NHEJ repair.
(PPTX)

S6 Fig Co-localization of *Pax3* and *Foxo1* in Foxo1-inv^+/+^ and wild type myoblasts. FISH analysis of Foxo1-inv^+/+^ (top left) and wild type myoblasts (top right) hybridized with BAC probes RP23–260F1 (green, *Pax3*) and RP24–391O12 (red, *Foxo1*). The table underneath shows the frequency of locus co-localization.
(TIF)

S7 Fig Enrichment of t(1;3) myoblasts from pools of CRISPR-Cas9 treated Foxo1-inv+/+/Ink4a-ARF-/- myoblasts. PCR analysis of pools, originated from ~30 cells of Cas9/RH30-like transduced Foxo1-inv+/+/Ink4a-ARF-/- myoblasts, 96-well plate #1. Numbers and letters indicate the position of the pool in the 96-well plate; m—1kb+ marker: position H12 corresponds to DNA from the original non-enriched Cas9/RH30-like transduced Foxo1-inv+/+/Ink4a-ARF-/- myoblasts.
(TIF)

S1 Table PAX3-FOXO1 target genes in ARMS. List of genes reported to be regulated by PAX3-FOXO by comparing gene expression levels in ARMS versus ERMS tumors and by forced expression of PAX3-FOXO1 in MEFs, NIH3T3, SAOS2 and C2C12 as published in [2] and references therein. The last column lists the up or down regulated genes in 64% t(1;3)-positive mouse myoblasts. These numbers were obtained by dividing the number of mapped RNA-seq reads in 64% *Pax3-Foxo1*-positive (1G3) cell pool by those in the wild type (1H3) cell pool. 45% of the genes (bold script) are regulated in the direction published. ND = no reads detected.
(XLSX)

S2 Table PAX3-FOXO1 expression signature in *PAX3-FOXO1* transduced cells. List of genes regulated by PAX3-FOXO1 in the ERMS cell line RD transduced with a *PAX3-FOXO1* retrovirus as reported by [50]. The last column lists the up or down regulated genes in 64% t(1;3)-positive mouse myoblasts. These numbers were obtained by dividing the number of mapped RNA-seq reads in the 64% *Pax3-Foxo1*-positive (1G3) cell pool by those in the wild type (1H3) cell pool. 52% of the genes (bold script) are regulated in the direction published. ND = no reads detected.
(XLSX)

S1 Protocol Strains of *E. coli.* PCR primers and oligonucleotides. Identification of the translocation breakpoints in ARMS.
(DOCX)
