# Supplementary figures and images for: Modeling of the Human Alveolar Rhabdomyosarcoma Pax3-Foxo1 Chromosome Translocation in Mouse Myoblasts Using CRISPR-Cas9 Nuclease
Source: PLoS Genet. 2015 Feb 6;11(2):e1004951. doi: 10.1371/journal.pgen.1004951 (PMC4319822; doi:10.1371/journal.pgen.1004951)

## Slide 1
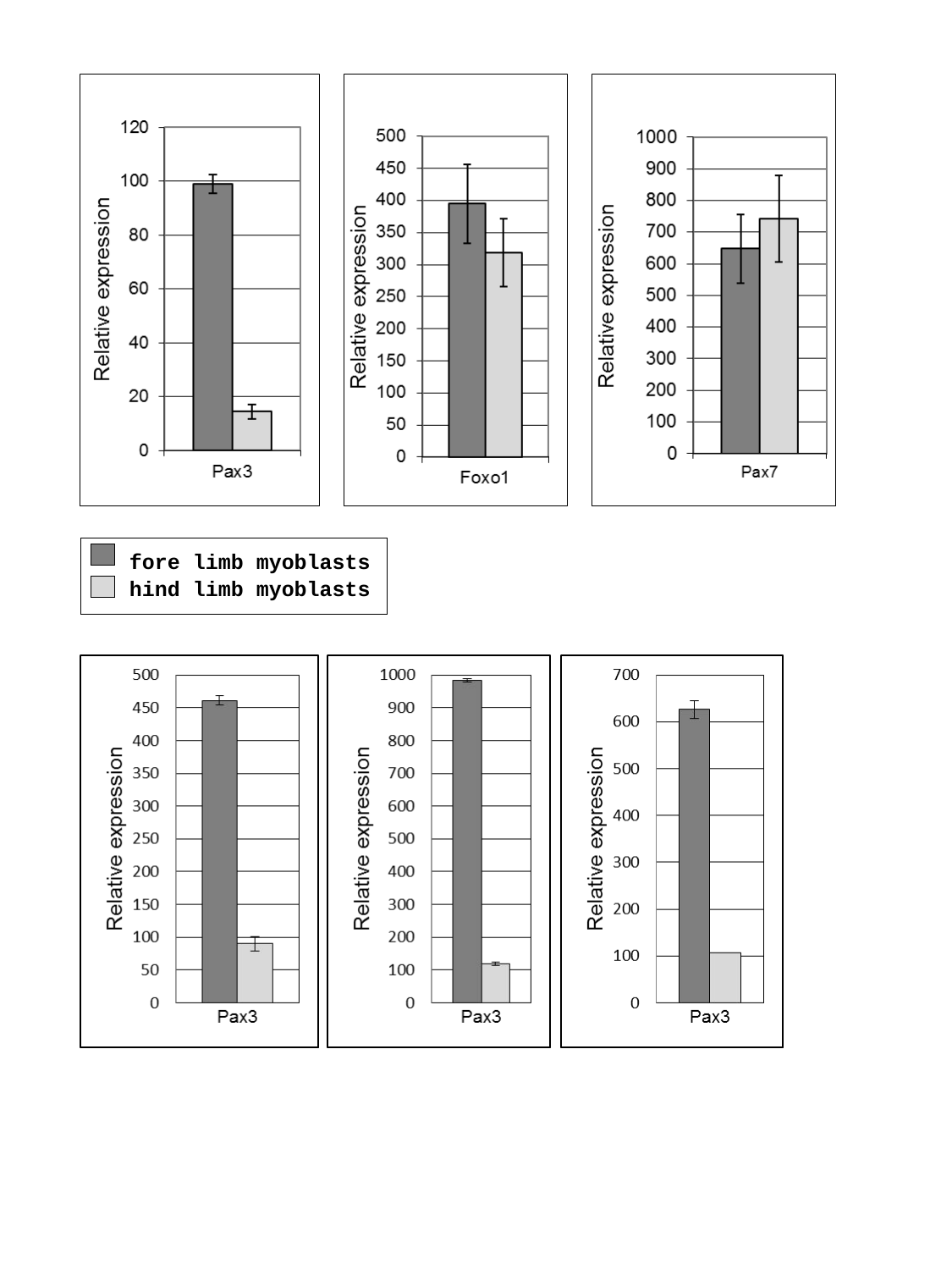

fore limb myoblasts
hind limb myoblasts

Supplement: S1 Fig — Upper 3 panels: Q-RT-PCR of a second fore and hind limb myoblast isolation experiment showing expression of Pax3, Foxo1 and Pax7 in both types of myoblasts. Lower 3 panels Q-RT-PCR showing the difference in Pax3 expression in fore and hind limb myoblasts in 3 additional, independent myoblast isolations. (PPT) [file pgen.1004951.s001.ppt]

## Slide 1
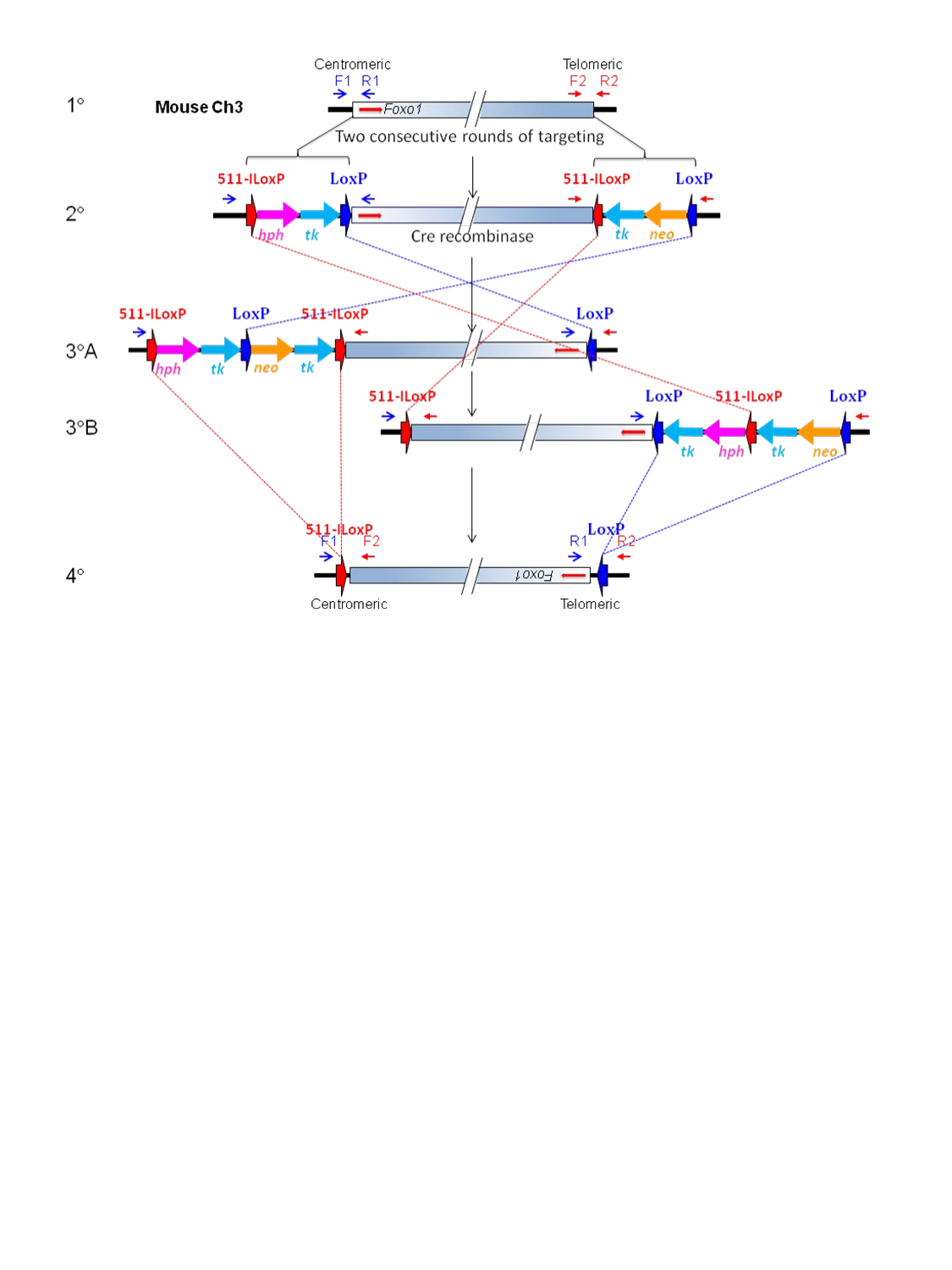

Supplement: S2 Fig — 1°—Schematic representation of the mouse/human 4.9 Mb syntenic region (gradient blue rectangle) on wild type mouse chromosome 3. The red arrow inside the gradient blue rectangle indicates the position and transcriptional direction of Foxo1. The arrows above the rectangle indicate the positions of the blue F1 centromeric and the red F2 telomeric forward primers and the blue R1 centromeric and red R2 telomeric reverse primers. 2°—Mouse chromosome 3 after consecutive centromeric and telomeric targeting of the borders of the 4.9 Mb syntenic region. Arrows underneath 511-ILoxP and LoxP—two incompatible loxP sites, indicate their relative orientation; hph—Hygromycin B resistance gene; neo—G418/Neomycin resistance gene; tk—HSV1-thymidine kinase gene. 3°A—First step of Cre-induced inversion of the double-targeted 4.9 Mb region. If the LoxP sites are used, the inversion of the 4.9Mb fragment places one LoxP site, the tk genes, and the antibiotic resistance genes between head-to-tail oriented 511-ILoxP sites. 3°B—Alternative first step of Cre-induced inversion of the double-targeted 4.9 Mb region. If the 511-ILoxP sites are used, the inversion of the 4.9Mb fragment places one 511-ILoxP site, the tk genes, and the antibiotic resistance genes between tail-to-head oriented LoxP sites. 4°—Cre-induced recombination via the 511-ILoxP (3°A) or the LoxP (3°B) sites results in the irreversible inversion of 4.9M syntenic region and flips the transcriptional orientation of Foxo1a. (PPT) [file pgen.1004951.s002.ppt]

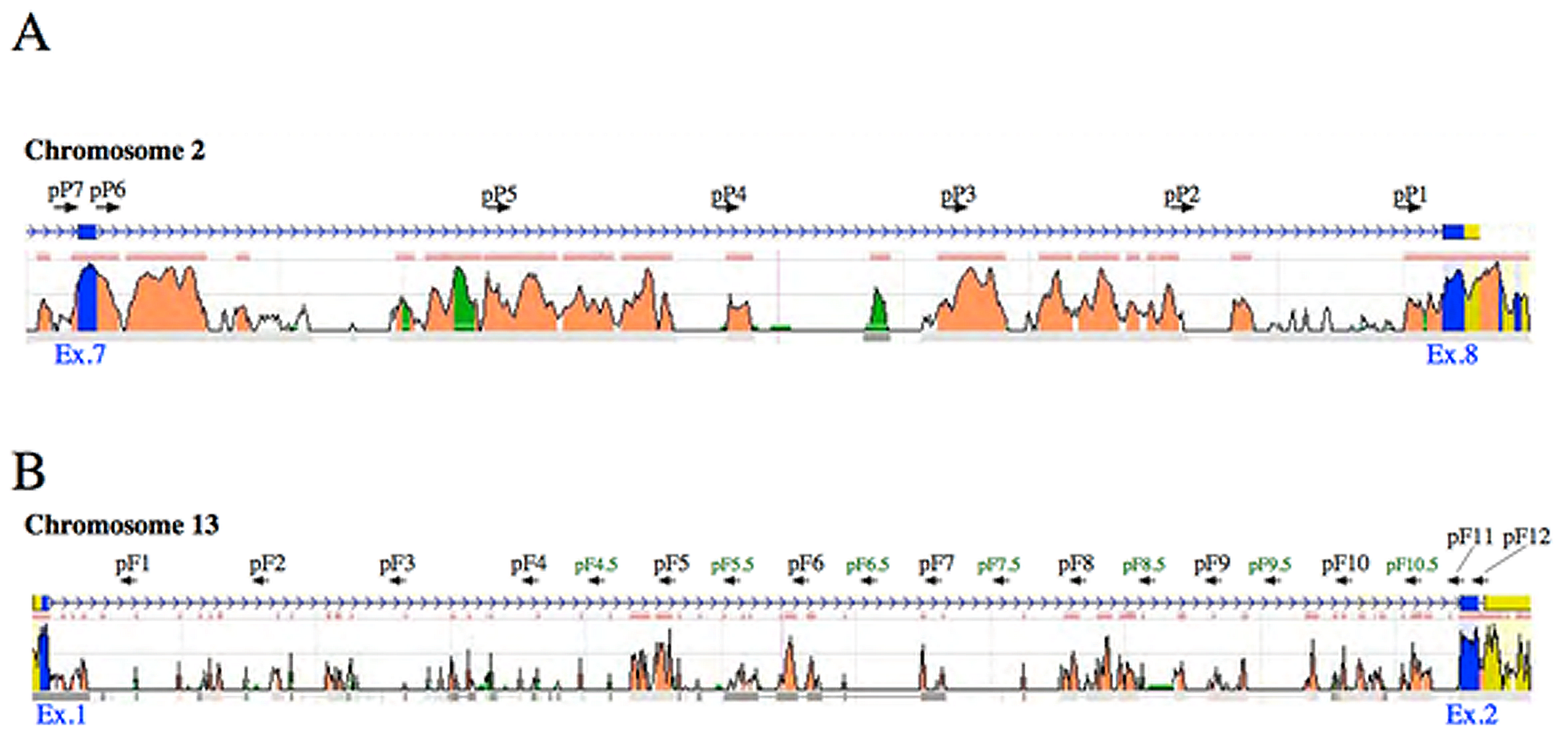

Supplement: S3 Fig — The human sequence of PAX3 intron 7 (A) and FOXO1 intron 1 (B) was compared to the mouse sequence using the ECR Browser genome analysis tool to show the location of evolutionarily conserved regions within these loci. Sequence comparison was performed with an ECR window of 100bp with a minimum similarity of 70%. The position of the exons (blue boxes) is indicated as well as the 5’ to 3’ direction of the gene (blue arrows). Blue peaks correspond to coding exons, yellow peaks correspond to 5’ or 3’ untranslated regions, orange peaks correspond to intronic non-coding conserved sequences and green peaks correspond to repetitive sequences. Primer positions are indicated (black arrows) with their names shown above. The PAX3 primers PAXLD1–7 are designated pP1–7. Primers PAXLD1–6 were designed 3kb apart from each other to span the PAX3 intron 7. Primer PAXLD7 is located immediately upstream of PAX3 exon 7. The FOXO1 primers FOXLD1–12 are designated pF1–12. Primers FOXLD1–11 are located 10kb apart from each other. Additional primers were designed between primers FOXLD4–11 to reduce the primer interval to 5kb. Primer FOXLD12 is located immediately downstream of exon 2. (TIF) [file pgen.1004951.s003.tif]

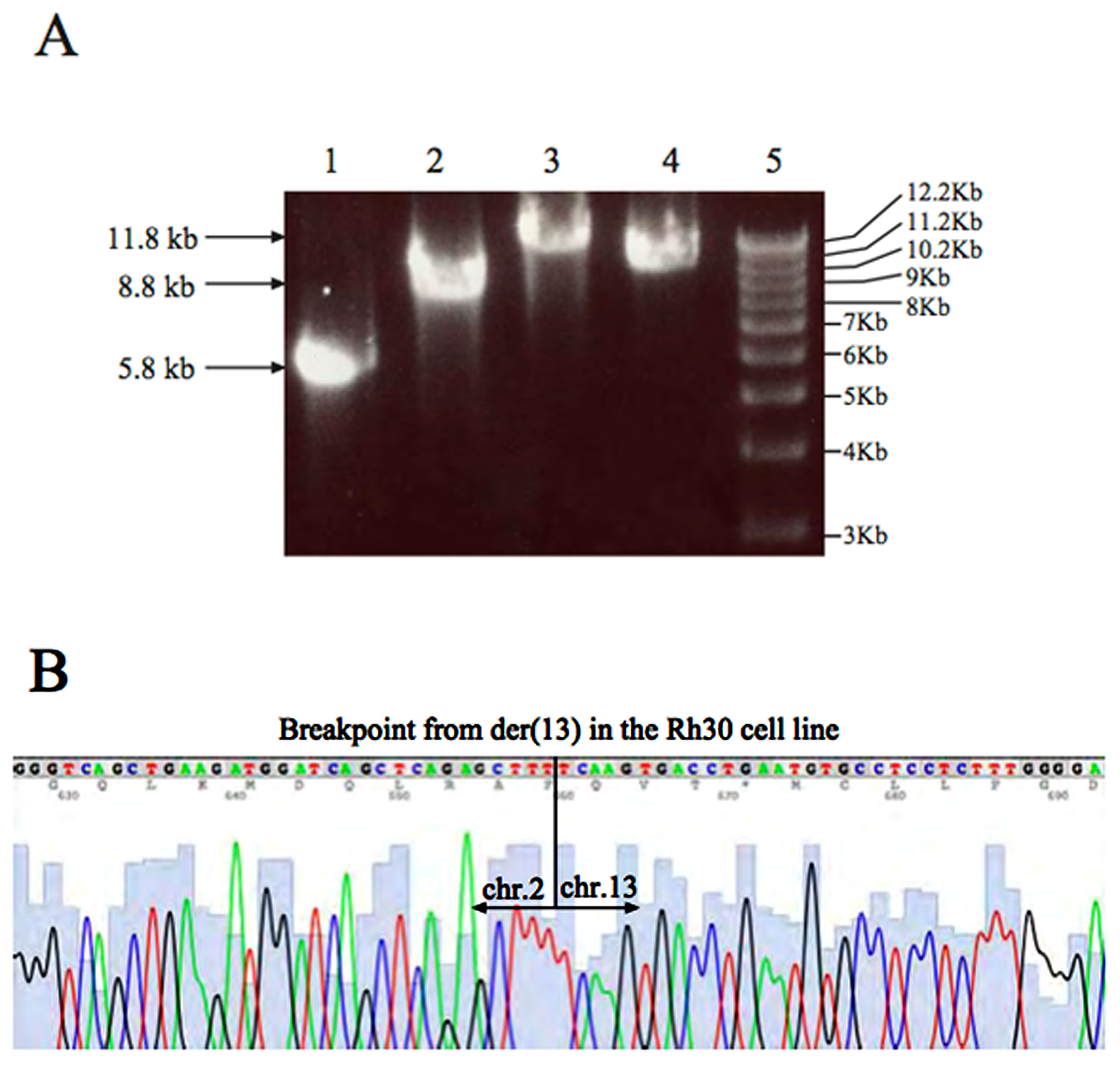

Supplement: S4 Fig — (A) Gel electrophoresis of LD-PCR products obtained in the amplification across the breakpoint in the Rh30 cell line. Lanes 1–3: reverse primer FOXLD6 in combination with PAXLD2, PAXLD3 and PAXLD4 generated fragments of 5.8kb, 8.8kb and 11.8kb, respectively. Lane 4: primers FOXLDwt and FOXLD8 resulting in a 10.1kb long fragment (positive control). Lane 5: 1kb DNA ladder (Invitrogen). (B) Sequences flanking the breakpoint in the Rh30 cell line showing the seamless transition between chromosomes 2 and 13. (TIF) [file pgen.1004951.s004.tif]

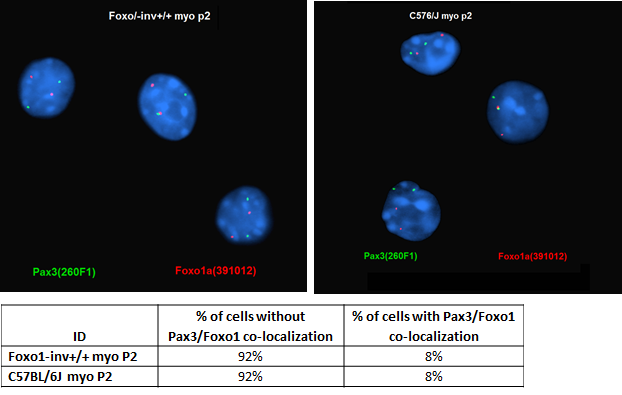

Supplement: S6 Fig — FISH analysis of Foxo1-inv+/+ (top left) and wild type myoblasts (top right) hybridized with BAC probes RP23–260F1 (green, Pax3) and RP24–391O12 (red, Foxo1). The table underneath shows the frequency of locus co-localization. (TIF) [file pgen.1004951.s006.tif]

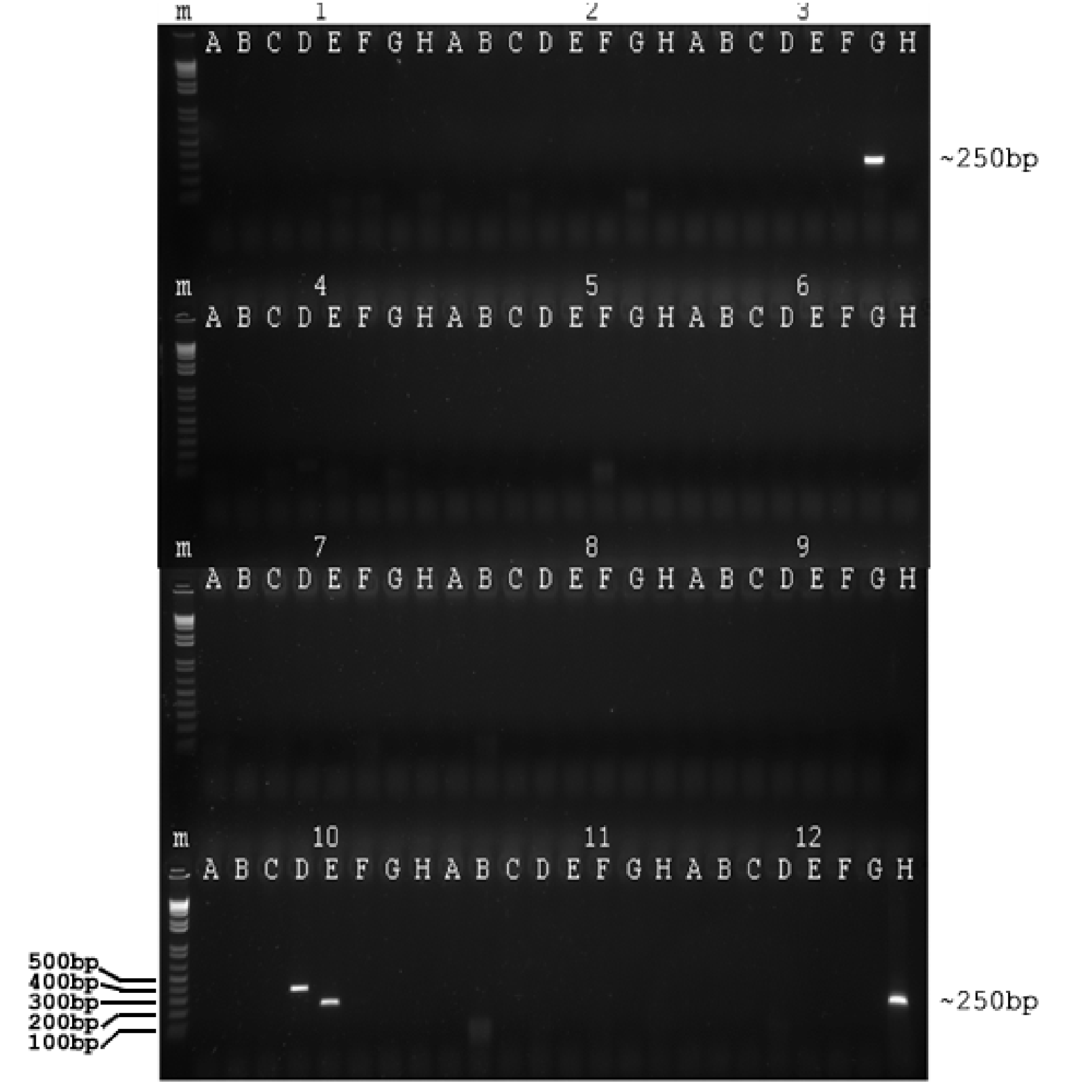

Supplement: S7 Fig — PCR analysis of pools, originated from ~30 cells of Cas9/RH30-like transduced Foxo1-inv+/+/Ink4a-ARF-/- myoblasts, 96-well plate #1. Numbers and letters indicate the position of the pool in the 96-well plate; m—1kb+ marker: position H12 corresponds to DNA from the original non-enriched Cas9/RH30-like transduced Foxo1-inv+/+/Ink4a-ARF-/- myoblasts. (TIF) [file pgen.1004951.s007.tif]
