# Supplementary material for: Modeling of the Human Alveolar Rhabdomyosarcoma Pax3-Foxo1 Chromosome Translocation in Mouse Myoblasts Using CRISPR-Cas9 Nuclease
Source: PLoS Genet. 2015 Feb 6;11(2):e1004951. doi: 10.1371/journal.pgen.1004951 (PMC4319822; doi:10.1371/journal.pgen.1004951)
Supplement: S5 Fig — Three additional fore limb myoblast Pax3-Foxo1 fusion sequences are shown below the predicted Pax3-Foxo1 fusion sequence. These were generated in an independent experiment producing sequences that are distinct from those depicted in Fig. 5E. Nucleotides in lower case represent Pax3 sequences, capitals represent Foxo1 sequences. Nucleotides in red lower case have been added randomly via NHEJ repair. (PPTX) [file pgen.1004951.s005.pptx]

## Slide 1
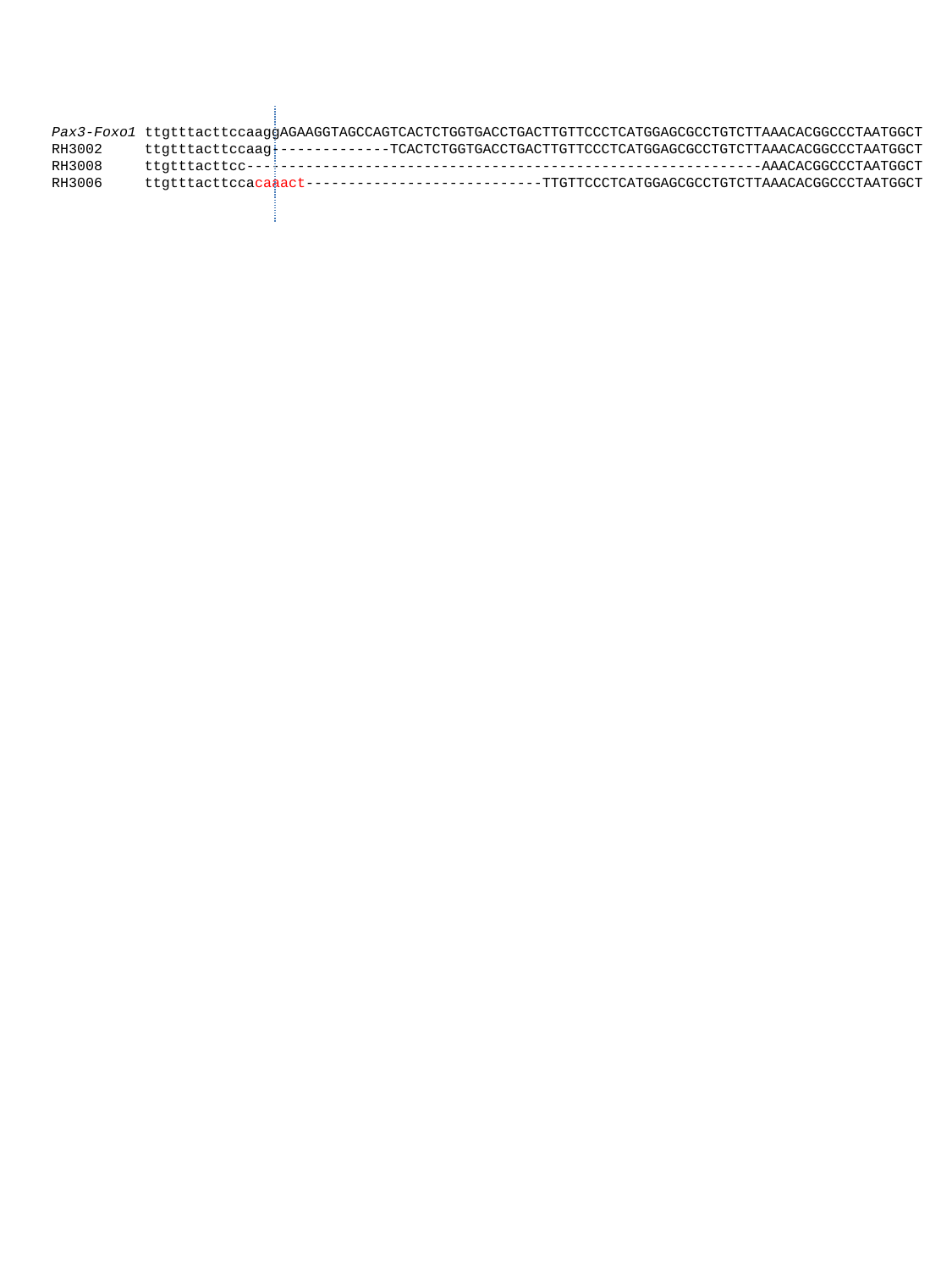

Pax3-Foxo1 ttgtttacttccaaggAGAAGGTAGCCAGTCACTCTGGTGACCTGACTTGTTCCCTCATGGAGCGCCTGTCTTAAACACGGCCCTAATGGCT
RH3002 ttgtttacttccaag--------------TCACTCTGGTGACCTGACTTGTTCCCTCATGGAGCGCCTGTCTTAAACACGGCCCTAATGGCT
RH3008 ttgtttacttcc-------------------------------------------------------------AAACACGGCCCTAATGGCT
RH3006 ttgtttacttccacaaact----------------------------TTGTTCCCTCATGGAGCGCCTGTCTTAAACACGGCCCTAATGGCT
