# Supplementary material for: Modeling of the Human Alveolar Rhabdomyosarcoma Pax3-Foxo1 Chromosome Translocation in Mouse Myoblasts Using CRISPR-Cas9 Nuclease
Source: PLoS Genet. 2015 Feb 6;11(2):e1004951. doi: 10.1371/journal.pgen.1004951 (PMC4319822; doi:10.1371/journal.pgen.1004951)
Supplement: S1 Protocol — Identification of the translocation breakpoints in ARMS. (DOCX) [file pgen.1004951.s010.docx]

Modeling of the human alveolar rhabdomyosarcoma ***Pax3-Foxo1*** chromosome translocation in mouse myoblasts using CRISPR-Cas9 nuclease.

**Irina V. Lagutina^1^, Virginia Valentine^2^, Fabrizio Picchione^1^,** Frank Harwood^1^, **Marcus B. Valentine^2^, Barbara Villarejo-Balcells^3^, Jaime J. Carvajal^3, 4^ ,**  **and Gerard C. Grosveld^1, *^**

**Author Affiliations:**

^1^Departments of Genetics and ^2^Tumor Cell Biology, St. Jude Children’s Research Hospital, Memphis, Tennessee 38105, USA

^3^ Division of Cancer Biology. The Institute of Cancer Research, London SW3 6JB, UK.

^4^ Centro Andaluz de Biología del Desarrollo (CSIC/UPO/JA), 41013 Sevilla, Spain.

***To whom correspondence should be addressed**

Gerard C. Grosveld,

Department of Genetics,

St Jude Children’s Research Hospital,

262 Danny Thomas Place,

Memphis, TN 38105, USA

Phone: 901-595-2279

Fax: 901-595-6035

e-mail: [gerard.grosveld@stjude.org](mailto:gerard.grosveld@stjude.org)

**PROTOCOL S1**

**Strains of *E. coli***

TOP10 (F– mcrA Δ(mrr-hsdRMS-mcrBC) Φ80lacZΔM15 ΔlacX74 recA1 araD139 Δ(ara leu) 7697 galU galK rpsL (StrR) endA1 nupG); DH10B™ (F– *mcr*A Δ(*mrr*-*hsd*RMS-*mcr*BC) Φ80*lac*ZΔM15 Δ*lac*X74 *rec*A1 *end*A1 *ara*D139 Δ(*ara leu*) 7697 *gal*U *gal*K *rps*L *nup*G λ–); **SW102**F- (*mcr*A Δ(*mrr-hsd*RMS-*mcr*BC) Φ80d*lac*Z M15 Δ*lac*X74 *deo*R *rec*A1 *end*A1 *ara*D139 Δ(*ara*, *leu*) 7649 *gal*U Δ*gal*K *rsp*L *nup*G [ *λcI*857 (*cro-bio*A) <> *tet*])

**PCR Primers and oligonucleotides**

The following oligonucleotides were used in this paper:

511-ILoxP-Not: GGCCGCATAACTTCGTATAATGTATACTATACGAAGTTATC

511-ILoxP-Not-C: GGCCGATAACTTCGTATAGTATACATTATACGAAGTTATGC

TK-511-ILoxP: CCGGGAGATGGGGGAGGCTAACTGACGGCAATAAAAAGACAGAATAAAACGCACGGGTGTTGGGTCGTTTGTTCATAACTTCGTATAATGTATACTATACGAAGTTATC

TK-511-ILoxP-C: CCGGGATAACTTCGTATAGTATACATTATACGAAGTTATGAACAAACGACCCAACACCCGTGCGTTTTATTCTGTCTTTTTATTGCCGTCAGTTAGCCTCCCCCATCTC

EM7-Neo-C: AGCGGCCGGAGAACCTGCGTGCAATCCATCTTGTTCAATGGCCGATCCCATGGTGGCCCTCCTATAGTGAGTCGTATTATACTATGCCGATATACTATGCCGATGATTAATTGTCAAACAGTCAG

TK-EM7: GCATATTAAGGTGACGCGTGTGGCCTCGAACACCGAGCGACCCTGCAGCGCTGACTGTTTGACAATTAATCATCGGCATAGTATATCGGCATAGTATAATACGACTCACTATAGGAGGGCCACC

5-cent-s: GGCCGCCCCTTCTGATACATTGTAAGCACTGGGTAAAAACATCGGTCTATAACATGGACCCTGATACTGAATAGCCCATGTCTTAGGACAAAGACTTTGAAACTTTGACA

5-cent-s-C: GGCCTGTCAAAGTTTCAAAGTCTTTGTCCTAAGACATGGGCTATTCAGTATCAGGGTCCATGTTATAGACCGATGTTTTTACCCAGTGCTTACAATGTATCAGAAGGGGC

3-cent: AGCTTAATGAACAGTTTATTTGTTTAGCTGCCTCTTGAGATTAGGATCCTTTAGTTCTTAAACGGAATTCGAGAAGGCCATCCAAACCTTCTAAAGAGCAGCCGGCTCTTTGGCCAATGCTCTGCTACAATAACAACATATCACAGATGG

3-cent-C: TCGACCATCTGTGATATGTTGTTATTGTAGCAGAGCATTGGCCAAAGAGCCGGCTGCTCTTTAGAAGGTTTGGATGGCCTTCTCGAATTCCGTTTAAGAACTAAAGGATCCTAATCTCAAGAGGCAGCTAAACAAATAAACTGTTCATTA

5-tel-s: GGCCGCCTAGGCTAAATTCAGTCTCATCTTTCAATGGCAGATACAGAGGTAGGAGCTGAAATCTGGGATAACGGAAGATGGTTATGAAATGGAAATGTAGTGTTTTGCTT

5-tel-s-C: CTAGAAGCAAAACACTACATTTCCATTTCATAACCATCTTCCGTTATCCCAGATTTCAGCTCCTACCTCTGTATCTGCCATTGAAAGATGAGACTGAATTTAGCCTAGGC

3-tel: AGCTTTGTTTTGTTTATTAGTGAAAACCAACTGTTAAATGTTTGAACATTTAATATGTCTTCTAAAATGTATCCAGAGAATACCATTTTCTAGAAATGTGCTATGCAGGACTTGGAAATGTTTTACAATAATTAAAATACTGATTTTACG

3-tel-C: TCGACGTAAAATCAGTATTTTAATTATTGTAAAACATTTCCAAGTCCTGCATAGCACATTTCTAGAAAATGGTATTCTCTGGATACATTTTAGAAGACATATTAAATGTTCAAACATTTAACAGTTGGTTTTCACTAATAAACAAAACAA

Foxo1 RH30 EcoNI:

TGACTGGCTACCTTCTCAGGTTTTAGAGCTAGAAATAGCAAGTTAAAATAAGGCTAGTCCGTTATCAACTTGAAAAAGTGGCACCGAGTCGGTGCTTTTT

Foxo1 RH30 EcoNI C:

AAAAAAGCACCGACTCGGTGCCACTTTTTCAAGTTGATAACGGACTAGCCTTATTTTAACTTGCTATTTCTAGCTCTAAAACCTGAGAAGGTAGCCAGTC

Pax3 RH30 AgeI:

CCGGGGAAAGTTTGTGTAACCTGTTTTAGAGCTAGAAATAGCAAGTTAAAATAAGGCTAGTCCGTTATCAACTTGAAAAAGTGGCACCGAGTCGGTGCTTTTTTT

Pax3 RH30 AgeI C:

CCGGAAAAAAAGCACCGACTCGGTGCCACTTTTTCAAGTTGATAACGGACTAGCCTTATTTTAACTTGCTATTTCTAGCTCTAAAACAGGTTACACAAACTTTCC

The following PCR and RT primers were used in this paper:

RP23-F: Gcagccaaaggtttagagg

RP23-TK-R: CCAATACGGTGCGGTATC

RP23-R: ctcaggttcagcacttgtc

RP23-NEO-F: CCCAGTCATAGCCGAATAG

RP24-F: ctgtggccctgtgcatatcg

RP23-F2: gtgactgggccataaagctc

RP24-R: cagggtccacctgcaagttc

RP23-R2: cctgcttgggtcatctgttc

RP24-hygro-R: ATCCACGCCCTCCTACATCG

RP24-tk-F: CGGGCTACTTGCCAATACGG

pTARBAC1-3F: CCATCCGGCTTACGATACTG

pTARBAC1-3R: CACTGAGGCGGCATATAGTC

pTARBAC1-5F: GCCGCTAATACGACTCAC

RP24-5R: CCCTCCATGTCCTATGTATC

mU6F: TGATCCGACGCCGCCATCTC

mU6R: CCTTAACAAGGCTTTTCTCCAAGGGATA

PAX3-RH30F: ATCTTGTGGCCCATTAGAGG

PAX3-RH30R2: CTAAGGGCTCTATCACAG

FOXO1-RH30F: CGGGTGGGCTTGTATAGAAC

FOXO1-RH30R: CTCTCAGACGCTCTCATTAAGG

RT Cnr1 Fwd: TGTACATTCTCTGGAAGGCTCACA

RT Cnr1 Rev: GGGTTCCACGCTGGATCA

RT Fgfr4 Fwd: AGGTTGGTGCTCGGAAAGC

RT Fgfr4 Rev: CACGAACCACTTGCCCAAA

mGapdhFwd: TCTTGTGCAGTGCCAGCCTC

mGapdhRev: CAAGAGAGTAGGGAGGGCTC

FOXLD1: AATACCTACAAAAGAATTTCTGTGCCACTGACTTG

FOXLD10.5: AAAAAGACTGCAGGAGACATAATAAGGAAATC

FOXLD10: TAAAAGTCACTCATCACAAACTTCCTGTTTAAAG

FOXLD11: GTACAATACTTCTCTGCTTGCAAAACTTCCTATTC

FOXLD12: ATGGCAAGTTACTGTGTTCCTCGCTTTTAAG

FOXLD2: CAAAGAATGCAATGGCACAAACTTTAACATTC

FOXLD3: TCCCTTCTATCTGAAAAATCTTCGAAAATAAAAC

FOXLD4.5: TAATGGAACATGGAATTACAATTTTCAAGGAG

FOXLD4: AAAACCTGGGAGGAGATCACAGATTCAAAGTC

FOXLD5.5: TCGGCCTCTGAAACACTTAGACTATTTAAAC

FOXLD5: AAAAATTCAGCTGAAGGATCTTTCTCAACAGTAG

FOXLD6.5: AGAGGCCAAAAGTGTATTCATTTAACATTATAGACAG

FOXLD6: TATTCAAACTCACGCCTAAAGAAATTCTTCAG

FOXLD7.5: AGCAGAATGCATGGACATTAATTAACAAAAAC

FOXLD7: TAGGAGCACAGAAAAGTGTAAAATACCTCAAAGG

FOXLD8.5: TGGCACGTGTCTATAGTCCTAGCTATTCAG

FOXLD8: CACTAACATAAGTCAAATAATGAATGCTGCTG

FOXLD9.5: AAATGAGAACATGTTTGGAAAACATAAAACAG

FOXLD9: AAAGAAGGGGGAAAGAAGACAGGAGAAGAGTG

FOXLDwt: ATCTTTCCAGCTTCCTTTCCTACAGATTCCTCTAC

PAX3 LD1: GTATGAACAGATACAATTTCTCCCTCTTTTCATC

PAX3 LD2: GCCAAATGTAGGAAAATGTTAGTGCTGTATTC

PAX3 LD3: TGAGAAGAGTTAACAGCAGGTAATGTCATTCC

PAX3 LD4: TAATAATGGAAAACCCACGTGGAGACCTAAC

PAX3 LD5: ACACCTGTCCTATTTCATGGATAATCTACTGAGG

PAX3 LD6: AGTCCCGTGTTTCTAGACAGACGATTTGCTG

PAX3 LD7: TGGCCTAAAAGAAAACATGATGGTTGACAATC

PAXLDseq2: AATAAACTAGTGTATCAATTGAAG

PAXLDwt: AGTATGTTAAGCTCTTGCCATGAAAAACTCATTTC

PAX3R: GCCTGTGGTATATCACCTCGAATATG

The following qPCR primers and probes were used in this paper:

RTmPax3Fwd: GCAGTCAGAGACTGGAACATATGAA

RTmPax3Rev: GGGACAATAGGGCTGAGATGTG

RTmPax3Probe: AATGTGGACAGTCTGC

MFoxoRealT Fw: GCCTCACACATCTGCCATGA

MFoxoRealT Rev: ACAGAGGCACTTGTAAAGGTGTCTT

MFoxoRT Pr: CCGCTTGACCCCCGT

mPax7-F: GGCCAAACTGCTGTTGATTACC

mPax7-R: GCTTCATACGGCGCTGTGT

mPax7-P: CCAAAAACGTGAGCCTG

mMyoD-F TGGTTCTTCACGCCCAAAAG

mMyoD-R TCTGGAAGAACGGCTTCGAA

mMyoD-P TGAAGCTTAAATGACACTCTTCCCAACTGTCC

mMyf5-F: TGGCCACTGCCTCATGTG

mMyf5-R: TGCGCCGATCCATGGTA

mMyf5-P: TTGCAAGAGGAAGTCC

**Identification of the translocation breakpoints in ARMS**

Although previous research has shown that A-RMS translocations occur within intron 7 of *PAX3* and intron 1 of *FOXO1,* the precise translocation of breakpoints have not been fully characterized. Furthermore, previous studies of A-RMS breakpoints have focused on describing the chromosome 2 rearrangements and hence there is little information regarding the translocation breakpoints on chromosome 13. To address the lack of information regarding the precise location of the breakpoints in both chromosomes, we mapped the translocations in 6 different A-RMS cell lines using Long- Distance Polymerase Chain Reaction (LD-PCR).

LD-PCR can amplify fragments up to ~20kb. In brief, forward primers selected at ~3kb intervals and spanning *PAX3* intron 7 were used in combination with reverse primers at ~10kb intervals spanning *FOXO1* intron 1. When translocation breakpoints could not be amplified, additional primers at ~5kb intervals spanning regions of the *FOXO1* gene were used (Figure S3). The shortest amplified fragment was subsequently cloned into the pCR-TOPO2.1 vector and single colonies analyzed by digestion and sequencing. BLAST analysis of the sequences across the breakpoints was performed using the *Homo sapiens* build 36.3 to determine the position of the breakpoints on chromosomes 2 and 13.

Control amplifications were performed in the LD-PCR experiments to assess the quality of the DNA. Fragments corresponding to the wild type *FOXO1* locus were amplified with primer pairs *FOXLDwt* + *FOXLD8* (10.1kb) and *FOXLDwt* + *FOXxLD9* (20.1kb). The wild type *PAX3* locus was amplified with the primer pair *PAXLDwt* + *PAXLD1* (12.1kb).

The breakpoint in Rh30 was amplified using the forward primers *PAXLD2* to *PAXLD4* and the reverse primer *FOXLD6,* which generated fragments of ~5.8kb, ~8.8kb and ~11.8kb (Figure S4A), respectively. Sequencing of the 5.8kb fragment with *T7* and *M13_*reverse primer did not reach the translocation breakpoint. The breakpoint was sequenced with the primer *PAXLDseq2.* BLAST analysis of the sequences across the breakpoint showed that the translocation occurred seamlessly between chromosomes 2 and 13 (Figure S4B). In this chromosomal rearrangement, chromosome 2 is disrupted at position 222,776,735 of the *Homo sapiens* built 36.3 while chromosome 13 is disrupted at position 40,085,867. This corresponds to 16.3kb downstream of the splice donor of *PAX3* exon 7 and 52.8kb upstream of the splice acceptor of *FOXO1* exon 2.

Amplification of genomic DNA across the translocation breakpoints in ARMS cell lines was performed using the Expand Long Template PCR System as described by the manufacturer. Primers were designed to be around 35bp. Briefly, 65 – 95ng of genomic DNA were amplified in a final volume of 51μl containing 3.75units of enzyme mix, 0.5μM of each dNTP and 0.3μM of forward and reverse primers. A pre-heated, high magnesium buffer (Buffer 3, Roche) was used. The PCR conditions consisted of an initial denaturing step at 95 ̊C for 2min, followed by 10 cycles of 94 ̊C for 10sec, 65 ̊C for 30sec and 68 ̊C for 20min. This was followed by 25 cycles of 94 ̊C for 15sec, 65 ̊C for 30sec, 68 ̊C for 30sec and 68 ̊C (+10sec/cycle) for 20min. A final incubation step was performed at 68 ̊C for 7min.
